# Briarenols W–Z: Chlorine-Containing Polyoxygenated Briaranes from Octocoral *Briareum stechei* (Kükenthal, 1908)

**DOI:** 10.3390/md19020077

**Published:** 2021-01-31

**Authors:** You-Ying Chen, Yi-Lin Zhang, Gene-Hsiang Lee, Lun Kelvin Tsou, Mingzi M. Zhang, Hsing-Pang Hsieh, Jih-Jung Chen, Chou-Yuan Ko, Zhi-Hong Wen, Ping-Jyun Sung

**Affiliations:** 1 Department of Marine Biotechnology and Resources, National Sun Yat-sen University, Kaohsiung 804201, Taiwan; D065020004@student.nsysu.edu.tw; 2 National Museum of Marine Biology and Aquarium, Pingtung 944401, Taiwan; 610863012@gms.ndhu.edu.tw; 3 Graduate Institute of Marine Biology, National Dong Hwa University, Pingtung 944401, Taiwan; 4 Instrumentation Center, National Taiwan University, Taipei 106319, Taiwan; ghlee@ntu.edu.tw; 5 Institute of Biotechnology and Pharmaceutical Research, National Health Research Institutes, Miaoli 350401, Taiwan; kelvintsou@nhri.edu.tw (L.K.T.); hphsieh@nhri.edu.tw (H.-P.H.); 6 Institute of Molecular and Genomic Medicine, National Health Research Institutes, Miaoli 350401, Taiwan; zhangmz@nhri.edu.tw; 7 Department of Chemistry, National Tsing Hua University, Hsinchu 300044, Taiwan; 8 Biomedical Translation Research Center, Academia Sinica, Taipei 115202, Taiwan; 9 Faculty of Pharmacy, School of Pharmaceutical Sciences, National Yang Ming Chiao Tung University, Taipei 112304, Taiwan; jjungchen@nycu.edu.tw; 10 Division of Gastroenterology, Department of Internal Medicine, Kaohsiung Armed Forces General Hospital, Kaohsiung 802301, Taiwan; gastroenterokjy@gmail.com; 11 Institute of Medical Science and Technology, National Sun Yat-sen University, Kaohsiung 804201, Taiwan; 12 Institute of BioPharmaceutical Sciences, National Sun Yat-sen University, Kaohsiung 804201, Taiwan; 13 Chinese Medicine Research and Development Center, China Medical University Hospital, Taichung 404394, Taiwan; 14 Graduate Institute of Natural Products, Kaohsiung Medical University, Kaohsiung 807378, Taiwan

**Keywords:** *Briareum stechei*, briarane, briarenol, inflammation, iNOS, COX-2

## Abstract

*Briareum stechei* is proven to be a rich source of 3,8-cyclized cembranoids (briarane) with a bicyclo[8.4.0] carbon core. In the present study, four previously unreported briaranes, briarenols W–Z (**1**–**4**), along with solenolide A (**5**), briarenolide M (**6**), briaexcavatolide F (**7**), and brianolide (**8**), were isolated and characterized through spectroscopic analysis, and the absolute configuration of **8** was corroborated by a single-crystal x-ray diffraction analysis. Briaranes **2** and **5** were found to induce significant inflammatory activity in lipopolysaccharide (LPS)-induced RAW 264.7 mouse macrophage cells by enhancing the expression of the inducible nitric oxide synthase (iNOS) and cyclooxygenase-2 (COX-2) proteins.

## 1. Introduction

Since briarein A, a 3,8-cyclized cembranoid (briarane), was first reported from a Caribbean octocoral *Briareum asbestinum* (Pallas, 1766) in 1977 [1], hundreds of marine origin briarane diterpenoids with novel structures and extensive bioactivities have been obtained from various octocorals [2,3], of which octocorals belonging to the genus *Briareum* have been recognized as the most important source of briarane-type natural products [3]. In our previous studies, a series of interesting briarane-type diterpenoids, including briarenols A–T [4,5,6,7,8,9,10,11,12], were isolated from various octocorals belonging to the genera *Briareum*, *Junceella*, and *Ellisella*, collected off the waters of Taiwan, and an anti-inflammatory assay was employed to evaluate the activities of these compounds in reducing the release of inducible oxide synthase (iNOS) and cyclooxygenase-2 (COX-2) in an in vitro pro-inflammatory macrophage culture model.

**Figure 1:**
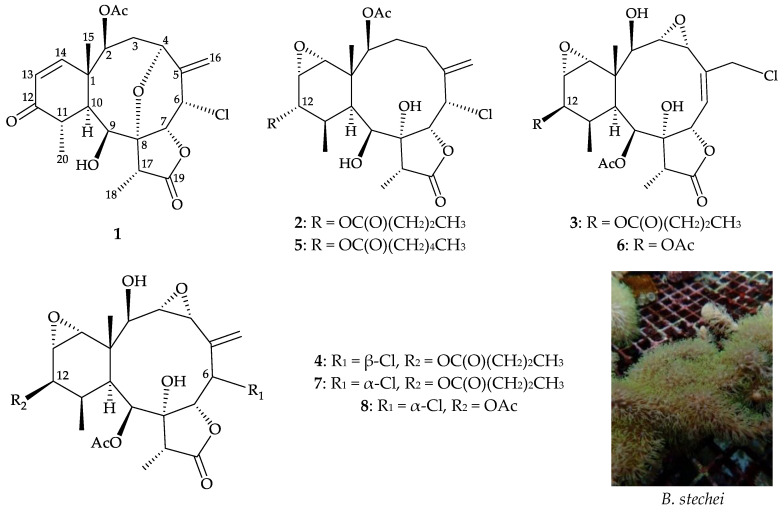
Structures of briarenols W–Z (**1**–**4**), solenolide A (**5**), briarenolide M (**6**), briaexcavatolide F (**7**), brianolide (**8**), and a picture of cultured *B. stechei*.

While most octocorals are classified as endangered species due to the exacerbating destruction of reef habitats, studies on the chemical constituents from cultured, potentially pharmaceutical, marine organisms have gained great attention [13]. Therefore, cultured octocoral *Briareum stechei* (Kükenthal, 1908) [14], originally dwelling in the waters of Taiwan, was selected for chemical investigation in our study. The results identified eight chlorine-containing briaranes, including four new compounds, briarenols W–Z (**1**–**4**), together with known briaranes, solenolide A (**5**) [15], briarenolide M (**6**) [16], briaexcavatolide F (**7**) [17], and brianolide (**8**) [18] (Figure 1). This study carried out the isolation and identification of the isolates as well as analyzing their bioactivity.

## 2. Results and Discussion

### 2.1. Chemical Identification of Isolated Briaranes

Freshly collected *B. stechei* were frozen and subsequently freeze-dried, powdered, and extracted with a mixture of methanol (MeOH) and dichloromethane (DCM) (1:1). Silica gel chromatography of the extract, followed by high performance liquid chromatography (HPLC), yielded briaranes **1**–**8**.

Briarenol W (**1**) was obtained as an amorphous powder. The positive mode electrospray ionization mass spectrum ((+)-ESIMS) showed a pair of peaks at *m/z* 461/463 ([M + Na]^+^/[M + 2 + Na]^+^) (3:1), with a relative intensity suggestive of a chlorine atom. NMR data coupled with the [M + Na]^+^ peak in the (+)high-resolution ESIMS ((+)-HRESIMS) at *m/z* 461.13377 suggested a molecular formula C_22_H_27_ClO_7_ (calculated for C_22_H_27_^35^ClO_7_ + Na, 461.13375) that indicated nine degrees of unsaturation. The IR spectrum indicated the presence of hydroxy (ν_max_ 3430 cm**^−^**^1^), γ-lactone (ν_max_ 1780 cm**^−^**^1^), ester carbonyl (ν_max_ 1733 cm**^−^**^1^), and α,β-unsaturated ketonic (ν_max_ 1670 cm**^−^**^1^) groups. The ^13^C NMR spectrum of **1** (Table 1) showed signals of 22 carbons. The multiplicity of the carbon signals was determined from the distortionless enhancement by polarization transfer (DEPT) and heteronuclear single quantum coherence (HSQC) spectra: four methyls, two methylenes (one olefin), ten methines (five bearing a heteroatom and two olefins), and six non-protonated carbons (three carbonyls, one olefin, and one bearing a heteroatom). From the ^13^C and ^1^H NMR spectra (Table 1 and Table 2), **1** was found to possess a γ-lactone (δ_C_ 175.3, C-19), an acetoxy (δ_H_ 2.15, 3H, s; δ_C_ 21.1, acetate methyl; δ_C_ 170.6, acetate carbonyl), an α,β-unsaturated ketonic (δ_H_ 6.95, 1H, d, *J* = 10.8 Hz, H-14; 5.95, 1H, d, *J* = 10.8 Hz, H-13; δ_C_ 205.6, ketonic carbonyl, C-12; 156.3, CH-14; 126.0, CH-13), and an exocyclic carbon–carbon double bond (δ_H_ 5.34, 1H, d, *J* = 1.8 Hz; 5.48, 1H, d, *J* = 1.8 Hz, H_2_-16; δ_C_ 137.5, C-5; 116.1, CH_2_-16) moieties. Five double bonds accounted for five unsaturated degrees. The remaining four degrees of unsaturation defined **1** as a tetracyclic molecule.

**Figure 2:**
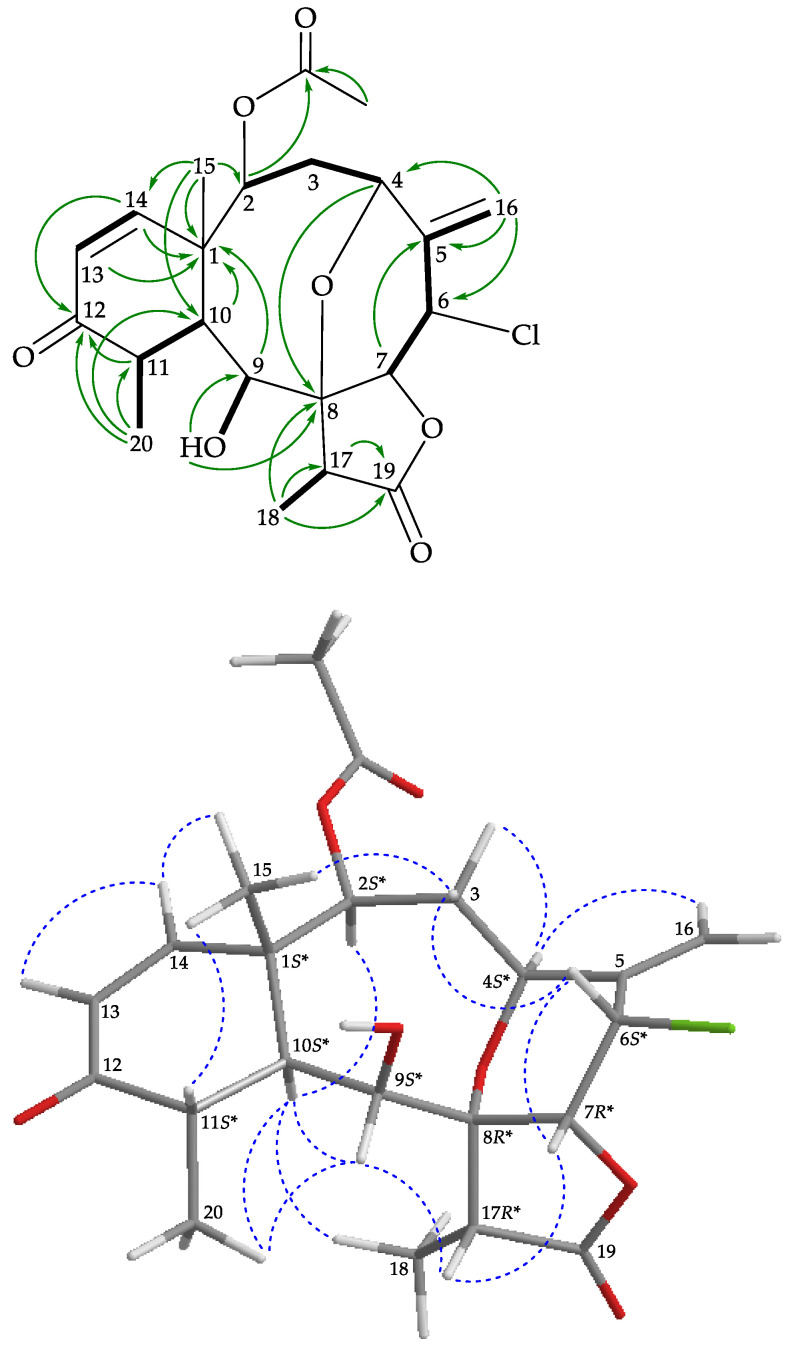
Key ^1^H–^1^H correlation spectroscopy (COSY) (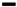), heteronuclear multiple bond coherence (HMBC) (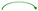), and protons with nuclear Overhauser effect spectroscopy (NOESY) (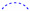) correlations for **1**.

The H-2/H_2_-3/H-4, H-6/H-7, H-10/H-11, H-13/H-14, H-11/H_3_-20, H-17/H_3_-18, and H-6/H_2_-16 (by allylic coupling) spin systems, measured in the ^1^H–^1^H correlation spectroscopy (COSY) (Figure 2), were fit to the regiochemistry of vicinal couplings in **1**. The fused tetracyclic network was established by heteronuclear multiple bond coherence (HMBC) experiments, particularly by the ^2^*J*- and ^3^*J*-^1^H–^13^C long-range correlations between protons and non-protonated carbons such as H-9, H-10, H-13, H-14, H_3_-15/C-1; H-7, H-16b/C-5; H-4, H_3_-18, OH-9/C-8; H-11, H-14, H_3_-20/C-12; and H-17, H_3_-18/C-19, thus permitted elucidation of the main carbon skeleton of **1** (Figure 2). The Me-20, Me-18, and Me-15 at C-11, C-17, and C-1 were confirmed by the HMBC correlations between H_3_-20/C-10, C-11, C-12; H_3_-18/C-8, C-17, C-19; and H_3_-15/C-1, C-2, C-10, C-14, respectively. An exocyclic double bond at C-5 was confirmed by the HMBC correlations between H_2_-16/C-4, C-5, and C-6. The hydroxy proton signal at δ_H_ 2.84 was revealed by its ^1^H-^1^H COSY correlation to H-9 (δ_H_ 4.53) and an HMBC correlation to C-9 (δ_C_ 76.9), indicating its attachment to C-9. The acetate ester at C-2 was established by a correlation between H-2 (δ_H_ 4.86) and the acetate carbonyl at δ_C_ 170.6, observed in the HMBC spectrum. The methine unit at δ_C_ 54.9 was more shielded than expected for an oxygenated C atom and correlated with the methine proton at δ_H_ 5.52 in the HSQC spectrum. This proton showed a ^3^*J*-correlation with H-7, in the ^1^H-^1^H COSY spectrum, confirming the attachment of a chlorine atom at C-6. An HMBC correlation between H-4 (δ_H_ 4.87), an oxymethine proton, and C-8, an oxygenated non-protonated carbon resonating at δ_C_ 82.4, suggested that the remaining oxygen atom had to be positioned between C-4 and C-8 to form an ether bridge in **1**.

The relative stereochemistry of **1** was established using a nuclear Overhauser effect spectroscopy (NOESY) experiment (Figure 2) and was found to be compatible with that of **1** offered by basic MMX study which justifies the NOESY experiments [19]. In naturally occurring briaranes, proton H-10 and Me-15 at C-1 are α- and β-oriented, respectively [3]. H-9, H-10, and H_3_-20 protons were proven to be located on the same face of the molecule. These protons, as a result of being correlated together, were assigned as α protons, as Me-15 was a β-substituent at C-1. Correlated with H-10, the H-2 proton had an α-orientation at C-2. Also, H_3_-18 was found to be associated with H-10, suggesting that the C-18 methyl in the γ-lactone moiety had an α-orientation. One of the methylene protons at C-3 (δ_H_ 3.32) exhibited a correlation with H_3_-15 and was assigned as H-3β, while the other was denoted as H-3α (δ_H_ 1.46). The correlations observed between H-3β/H-6, H-6/H-7, and H-7/H-17 reflected the β-orientation of both protons at C-6 and C-7. The *cis* geometry of the C-13/14 double bond was indicated by a 10.8 Hz coupling constant between H-13 (δ_H_ 5.95) and H-14 (δ_H_ 6.95), and further confirmed by a NOESY correlation between these two olefinic protons. Furthermore, H-3α showed a correlation with H-4, demonstrating the S*-configuration of stereogenic center C-4. The remaining stereogenic carbon, C-8, lacked a proton but there were correlations between H-9/H-17 and H-7/H-17, indicating that C-8 was in an R*-configuration, as evidenced by modeling analysis. Based on the above findings, the configuration of the stereogenic centers of **1** was assigned as (1S*,2S*, 4S*,6S*,7R*,8R*,9S*,10S*,11S*,17R*) (Supplementary Materials, Figures S1–S11).

Briarenol X (**2**) had the molecular formula C_26_H_37_ClO_9_ on the basis of (+)-HRESIMS at *m/z* 551.20198 (calculated for C_26_H_37_ClO_9_ + Na, 551.20183). The IR spectrum of **2** showed bands at 3484, 1777, and 1729 cm^−^^1^, consistent with the presence of hydroxy, γ-lactone, and ester carbonyl groups, respectively. ^13^C NMR and DEPT spectroscopic data (Table 1) revealed that **2** contained an exocyclic carbon–carbon double bond (δ_C_ 140.3, C-5; 118.6, CH_2_-16) and three carbonyl resonances (δ_C_ 176.4, 173.3, and 168.3). Two esters were identified as acetate and *n*-butyrate respectively by the presence of resonances in the ^1^H NMR spectrum of **2** at δ_H_ 2.19 (3H, s), 0.99 (3H, t, *J* = 7.2 Hz), 1.69 (2H, sext, *J* = 7.2 Hz), and 2.34 (2H, t, *J* = 7.2 Hz) (Table 2).

**Figure 3:**
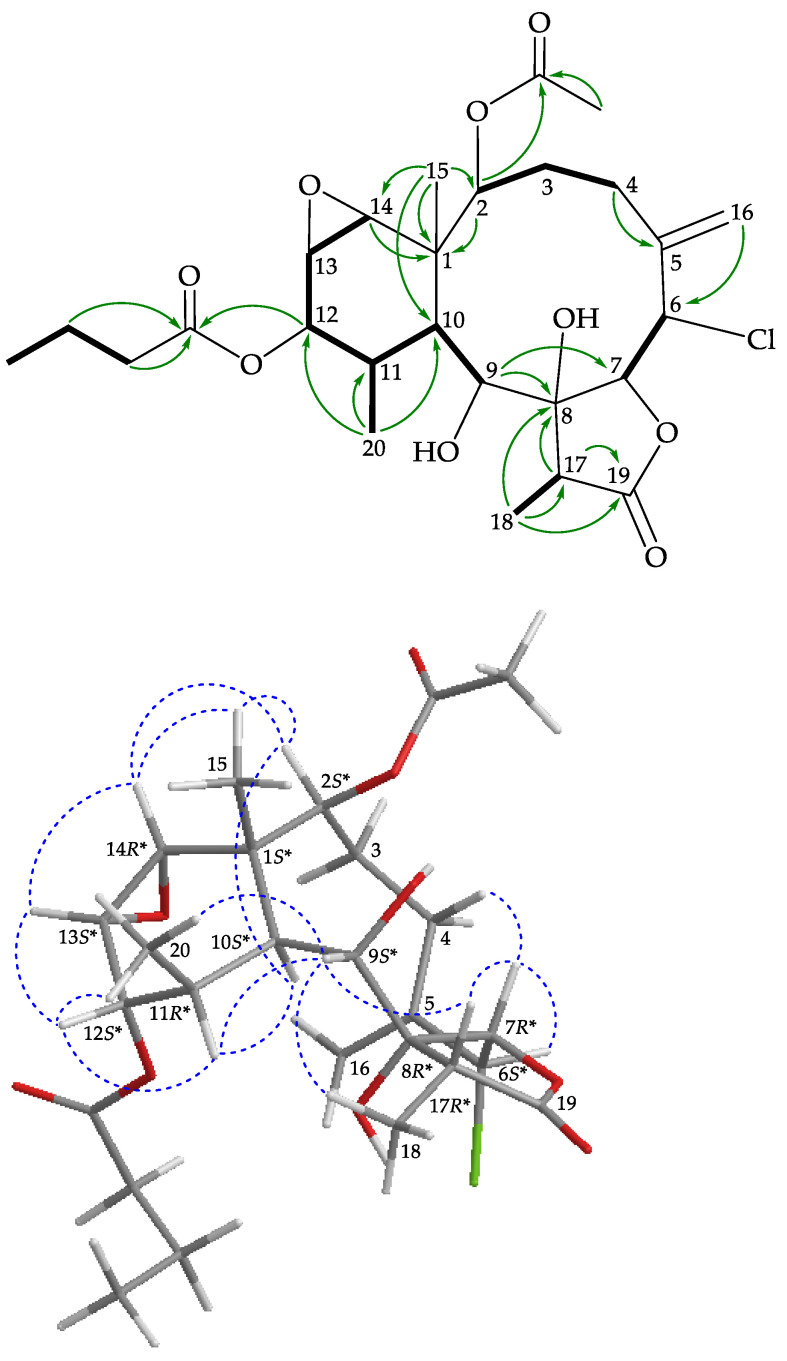
Key COSY (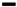), HMBC (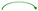), and protons with NOESY (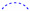) correlations for **2**.

In the HMBC spectrum of **2** (Figure 3), the *n*-butyrate positioned at C-12 was confirmed from the long-range coupling between H-12 (δ_H_ 4.74) with the carbonyl carbon (δ_C_ 173.3) of the *n*-butyroxy group. The HMBC correlation also revealed that one acetate was attached to C-2. These data, together with the other ^1^H-^13^C long-range correlations, unambiguously established the molecular framework of **2**. According to the above observations, metabolite **2** seemed to be very similar to solenolide A (**5**) [15], which was previously isolated from an octocoral *Solenopodium* sp. By means of 1D and 2D NMR data it was found that the *n*-hexanoate group at C-12 position in solenolide A (**5**) was replaced by an *n*-butyrate group in **2**.

The relative configuration of **2** was determined by NOESY analysis (Figure 3). The NOESY correlations between H-10/H-11 and H-10/H-2 required that all of these groups were in α-face and correlations of H_3_-15/H-14, H-14/H-13, and H-13/H-12 indicated β-disposition for these groups. The correlations between H-9/H-11, H-9/H_3_-18, and H-9/H-7 suggested that H-9 and H_3_-18 were α-oriented and H-7 was β-oriented from modeling analysis. A correlation between H-6 and H-7 reflected the β-orientation of protons at C-6. The negative optical rotation value of **2** ([α]${}_{D}^{20}$ −14 (*c* 0.05, CHCl_3_)) was similar to that of **5** (solenolide A) ([α]${}_{D}^{20}$ −28 (*c* 0.04, CHCl_3_); reference [15], [α]${}_{D}^{20}$ −56 (*c* 0.63, CHCl_3_)) that was also obtained in this study, suggesting that **2** and **5** had 1S*,10S*-configurations in the ring junction. Based on the above findings, the structure of **2**, including the relative configuration, was elucidated ambiguously, and its stereogenic centers were assigned as (1S*,2S*,6S*,7R*,8R*,9S*,10S*,11R*,12S*,13S*, 14R*,17R*) (Supplementary Materials, Figures S12–S22).

**Figure 4:**
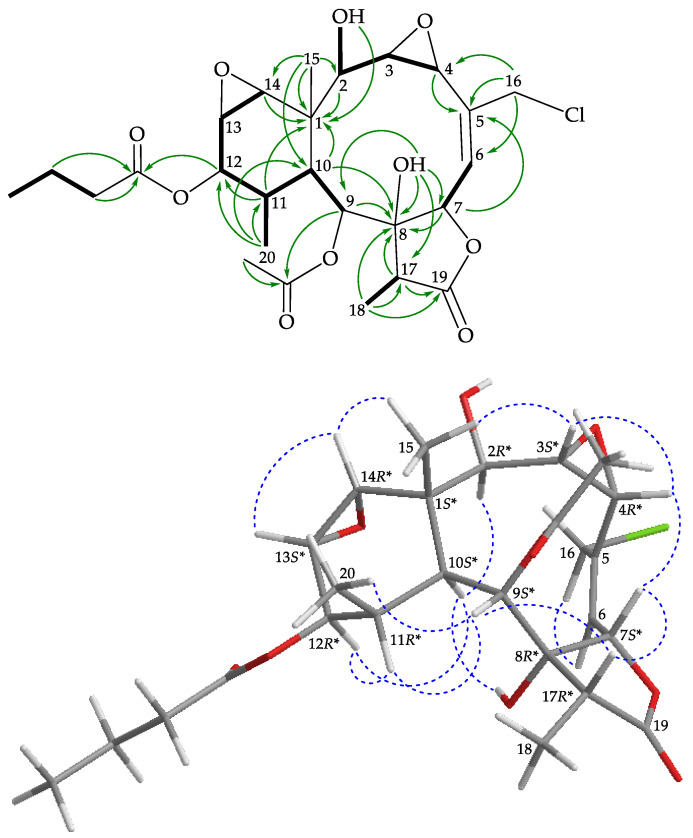
Key COSY (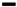), HMBC (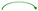), and protons with NOESY (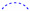) correlations for **3**.

Briarane **3** (briarenol Y) was isolated as an amorphous powder that showed two sodiated adduct ion peaks in (+)-HRESIMS at *m/z* 565.18106 [M + Na]^+^ and 567.17800 [M + 2 + Na]^+^ (3:1), which accounted for a chlorine atom in the molecular formula, C_26_H_35_ClO_10_ (calculated for C_26_H_35_^35^ClO_10_ + Na, 565.18110). Its absorption peaks in the IR spectrum showed ester carbonyl, γ-lactone, and broad OH stretching at 1738, 1778, and 3459 cm^–1^, respectively. From the ^13^C and ^1^H NMR (Table 1 and Table 2), three carbonyl resonances at δ_C_ 175.5 (C-19), 173.1, and 169.5 confirmed the presence of a γ-lactone and two ester groups; an acetate methyl (δ_H_ 2.22, 3H, s) and an *n*-butyrate (δ_H_ 2.35, 2H, t, *J* = 7.2 Hz; 1.67, 2H, sext, *J* = 7.2 Hz; 0.96, 3H, t, *J* = 7.2 Hz) were also observed. Two disubstituted epoxy groups were deduced from the signals of four oxymethine carbons at δ_C_ 62.1 (CH-14), 60.5 (CH-3), 58.1 (CH-4), and 57.2 (CH-13). The chemical shifts of oxymethine protons at δ_H_ 3.29 (1H, d, *J* = 3.6 Hz, H-14), 3.38 (1H, dd, *J* = 9.2, 4.0 Hz, H-3), 4.14 (1H, dd, *J* = 4.0, 2.0 Hz, H-4), and 3.19 (1H, dd, *J* = 3.6, 0.8 Hz, H-13) further confirmed the presence of these two groups. Based on the ^13^C NMR data and degrees of unsaturation, **3** was established as a pentacyclic diterpenoid. It was found that the ^1^H and ^13^C NMR data of **3** resembled those of a known briarane, briarenolide M (**6**) (Figure 1) [16], except that the signals corresponding to the C-12 acetoxy group in **6** were replaced by signals for an *n*-butyroxy group in **3**. Locations of the functional groups were confirmed by other HMBC and COSY correlations (Figure 4).

The relative configuration of **3** was determined from the NOESY spectrum (Figure 4), which showed NOESY correlations among the corresponding protons similar to those of **6** [16]. The negative optical rotation value of **3** ([α]${}_{D}^{24}$ −73 (*c* 0.1, CHCl_3_)) was similar to that of **6** ([α]${}_{D}^{25}$ −58 (*c* 0.7, CHCl_3_)) [16] in direction and magnitude, suggesting that **3** and **6** had 1S*,10S*-configurations in the ring junction. Thus, briarenol Y was assigned as the structure of **3** and the configurations of the stereogenic carbons were elucidated as (1S*,2R*,3S*,4R*,7S*,8R*,9S*, 10S*,11R*,12R*,13S*,14R*,17R*) (Supplementary Materials, Figures S23–S33).

**Figure 5:**
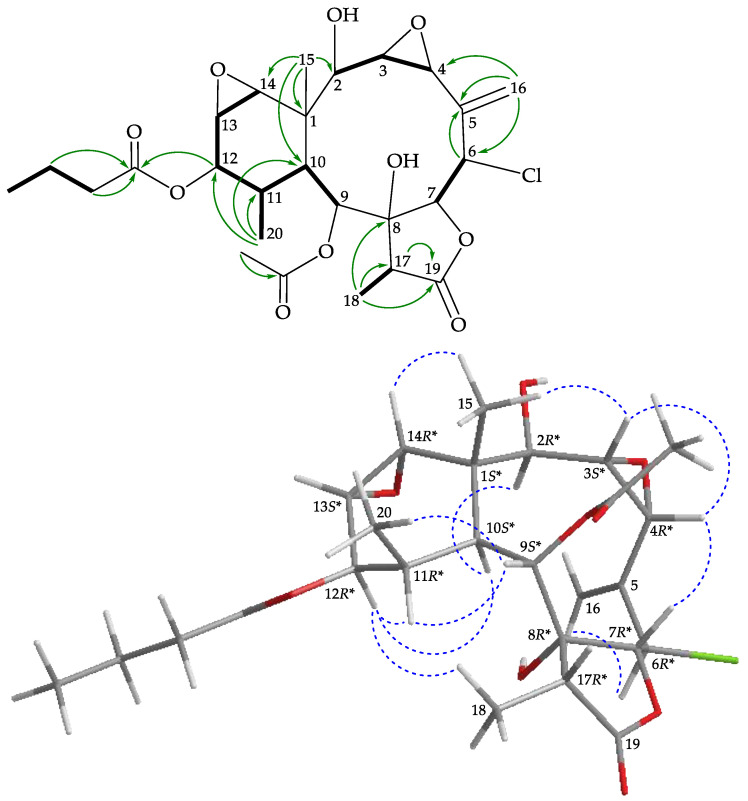
Key COSY (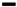), HMBC (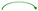), and protons with NOESY (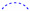) correlations for **4**.

Briarane **4** (briarenol Z) was isolated as an amorphous powder and had the molecular formula C_26_H_35_ClO_10_ on the basis of (+)-HRESIMS (see Materials and Methods section). The IR spectrum of **4** showed bands at 3450, 1777, and 1736 cm^–1^, consistent with the presence of hydroxy, γ-lactone, and ester carbonyl groups. It was found that the spectroscopic data of **4** were very similar to those of a known briarane metabolite, briaexcavatolide F (**7**) [17]. However, a comparison of the ^1^H and ^13^C NMR chemical shifts of C-6 methine (δ_H_ 4.92, 1H, d, *J* = 10.4 Hz; δ_C_ 63.2), C-7 oxymethine (δ_H_ 4.71, 1H, d, *J* = 10.4 Hz; δ_C_ 83.0), and C-5 sp^2^ non-protonated carbon (δ_C_ 137.4) of **4** (Table 1 and Table 2) with those of **7** (δ_H_ 5.83, 1H, d, *J* = 3.3 Hz; δ_C_ 61.8, CH-6; δ_H_ 5.73, 1H, d, *J* = 3.3 Hz; δ_C_ 79.8, CH-7; δ_C_ 138.7, C-5) [17] showed that H-6 in **4** was α-oriented. The NOESY spectrum exhibited a strong correlation from H-7 to H-4, but not with H-6 (Figure 5), and a large vicinal proton coupling constant (*J* = 10.4 Hz) was detected between H-7 and H-6, indicating that the dihedral angle between H-6 and H-7 was approximately 180°, that H-6 was α-oriented in **4**, and that this compound should possess a structure as represented by formula **4**. The structure of **4** was further confirmed by 2D NMR experiments (Figure 5) and its stereogenic centers were assigned as (1S*,2R*,3S*,4R*,6R*,7R*,8R*,9S*,10S*,11R*,12R*,13S*,14R*, 17R*) by NOESY experiment (Figure 5) (Supplementary Materials, Figures S34–S44).

Known briaranes **5**–**8** were found to be identical with briarenolide solenolide A [15], briarenolide M [16], briaexcavatolide F [17], and brianolide [18], respectively by comparison of the spectroscopic data with those reported previously.

**Figure 6:**
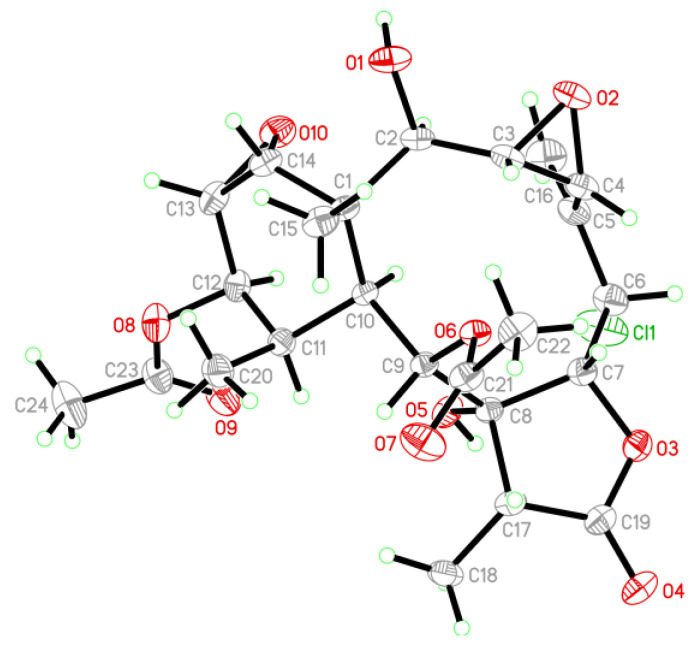
Oak Ridge Thermal Ellipsoid Plot (ORTEP) plot revealing the absolute configuration of **8** (methanol molecule has been omitted for clarify).

A single-crystal x-ray diffraction was used to confirm the structure of brianolide **8**. The Oak Ridge Thermal Ellipsoid Plot (ORTEP) diagram (Figure 6) showed that the absolute configurations of the stereogenic carbons of **8** were confirmed as (1*S*,2*R*,3*S*,4*R*,6*S*,7*R*, 8*R*,9*S*,10*S*,11*R*,12*R*,13*S*,14*R*,17*R*) (Flack parameter x = 0.095(8)).

### 2.2. Bioactivity of Isolated Briaranes

It is well documented that the microbial lipopolysaccharide (LPS) can activate toll-like receptor-4 (TLR-4), located in the mammal cell membrane surface, which triggers inflammatory responses through the activation of intracellular signal transduction and the upregulation of pro-inflammatory proteins inducible nitric oxide synthase (iNOS) and cyclooxygenase-2 (COX-2) [20]. It is well known that inhibition of the expression of pro-inflammatory proteins iNOS and COX-2 in LPS-stimulated macrophage cells can be used as for in vitro screening of anti-inflammatory compounds [21,22,23]. The massive production of inflammatory mediators, nitric oxide (NO) and prostaglandin E2 (PGE2) via pro-inflammatory proteins iNOS and COX-2, respectively, plays an important pathophysiological role in inflammation. There are two COX isozymes, COX-1 (cyclooxygenase-1) and COX-2, catalyzing the prostaglandin synthesis. COX-1 is constitutively expressed in normal physiological conditions. Unlike COX-1, COX-2 is an inducible enzyme that increases following injury or inflammation [24,25]. COX-2 plays a more vital role in pathology than COX-1 under inflammatory processions.

The effects of briaranes **1**–**7** on the release of iNOS and COX-2 from LPS-stimulated RAW 264.7 macrophage cells were assessed (Table 3). Briaranes **2** and **5** at 10 μM enhanced the release of iNOS (142.03 and 134.11%, respectively) and COX-2 (159.21 and 196.03%, respectively) as compared to results for the cells stimulated with LPS only. It is interesting to note that these findings seem to be contrary to results claimed to show that most briarane-type natural products from octocorals are anti-inflammatory [26]. Structure–activity relationships among these marine diterpenoids will be evaluated if enough materials are obtained.

## 3. Materials and Methods

### 3.1. General Experimental Procedures

A digital polarimeter (model P-1010; JASCO Corp., Tokyo, Japan) was used to determine optical rotations of the samples. IR spectra were collected using a spectrophotometer (model Nicolet iS5 FT-IR; Thermo Fisher Scientific, Waltham, MA, USA). ^1^H and ^13^C NMR spectra were recorded on ECZ-400 or ECZ-600 spectrometers (Jeol Ltd., Tokyo, Japan) for solutions in CDCl_3_ (with residual CHCl_3_ (δ_H_ 7.26 ppm) and CDCl_3_ (δ_C_ 77.0 ppm) as internal standards). For coupling constants (J), the results were given in frequency units (Hz). For positive mode ESIMS and HRESIMS, the results were obtained using a SolariX FTMS mass spectrometer (7 Tesla; Bruker, Bremen, Germany). The extracted samples were separated by column chromatography with silica gel (between 230 and 400 meshes; Merck). Thin-layer chromatography plates with silica gel coated with fluorescent indicator F_254_ were employed. For visualization, the plates were charred with 10% (*v/v*) aqueous sulfuric acid solution, then heated at 105 °C until spots were seen. For normal-phase HPLC separation, a system containing a pump (Hitachi model L-7110; Tokyo, Japan) and an injection interface (No. 7725; Rheodyne) was employed, equipped with a semi-preparative column with dimensions of 250 × 20 mm and a 5-μm particle size (Sigma). For reverse-phase HPLC separation, a system composed of a pump (Hitachi model L-2130) and a diode-array detector (LaChrom L-2455, Hitachi) was used, equipped with a column with dimensions of 2.1 × 25 cm and a 5-μm particle size (Phenomenex).

### 3.2. Animal Material

Specimens of *B. stechei* used for this study were collected from an 80-ton culturing tank equipped with a flow-through seawater system located in the National Museum of Marine Biology and Aquarium (NMMBA) in April 2016. Identification of the species of this organism was performed by comparison, as described in previous studies [14]. Living reference specimens are maintained in the authors’ marine organism culturing tanks and a voucher specimen was deposited with the NMMBA (voucher no.: NMMBA-TW-GC-2016-031), Taiwan.

### 3.3. Extraction and Isolation

Sliced bodies (wet/dry weight = 3980/1860 g) of the specimen were grounded and extracted with a mixture of MeOH and CH_2_Cl_2_ (1:1) to provide an extract (104 g). The extract was then applied to a silica gel column chromatography (Si C.C.) and eluted with gradients of *n*-hexane/EtOAc (stepwise from 50:1–1:2) to furnish fractions A–L. Fractions H and I were combined (19.0 g) and washed with acetone to obtain an undissolved material brianolide (**8**) (2.59 g). The dissolved material was separated with Si C.C. using *n*-hexane/EtOAc (stepwise from 50:1−pure EtOAc) to obtain fractions H1−H8. Fraction H6 was chromatographed with Si C.C. using *n*-hexane/EtOAc/acetone to obtain fractions H6A−H6K. Fraction H6E was separated by Si C.C. using a mixture of CH_2_Cl_2_ and acetone (4:1) to obtain fractions H6E1−H6E6. Fraction H6E1 was repurified by RP-HPLC using a mixture of MeOH and H_2_O (7:3) to yield fractions H6E1A–H6E1K. H6E1K was separated by RP-HPLC using a mixture of MeOH and H_2_O (8:2; at a flow rate = 4.0 mL/min) to yield briarenol X (**2**) (0.9 mg). Fraction H6E2 was separated by NP-HPLC using a mixture of CH_2_Cl_2_ and acetone (8:1) to yield fractions H6E2A−H6E2E. Fraction H6E2A was repurified by RP-HPLC using a mixture of MeOH and H_2_O (7:3) to yield fractions H6E2A1−H6E2A6. Fraction H6E2A5 was further separated by RP-HPLC with a mixture of MeOH and H_2_O (7:3) to yield fractions H6E2A5A−H6E2A5E. Fraction H6E2A5E was separated by RP-HPLC with a mixture of MeOH and H_2_O (8:2; at a flow rate = 4.0 mL/min) to yield solenolide A (**5**) (0.6 mg). Fraction H6E3 was repurified by NP-HPLC using a mixture of CH_2_Cl_2_ and acetone (9:1) to yield fractions H6E3A−H6E3G. Fraction H6E3D was separated by RP-HPLC using a mixture of MeOH and H_2_O (7:3; at a flow rate = 5.0 mL/min) to yield briarenols W (**1**) (0.6 mg), Y (**3**) (2.8 mg), and Z (**4**) (1.2 mg), respectively. Fraction H6E4 was separated by NP-HPLC using a mixture of CH_2_Cl_2_ and acetone (6:1) to obtain fractions H6E4A−H6E4I. Fraction H6E4E was repurified by NP-HPLC using a mixture of *n*-hexane and acetone (5:2) to obtain fractions H6E4E1−H6E4E10. Fraction H6E4E8 was separated by RP-HPLC with a mixture of MeOH and H_2_O (7:3; at a flow rate = 5.0 mL/min) to yield briarenolide M (**6**) (4.5 mg). Fraction H6F was separated by RP-HPLC with a mixture of MeOH and H_2_O (7:3; at a flow rate = 5.0 mL/min) to yield briaexcavatolide F (**7**) (8.9 mg).

Briarenol W (**1**): amorphous powder; [α]${}_{D}^{24}$ −29 (*c* 0.1, CHCl_3_); IR (KBr) ν_max_ 3430, 1780, 1733, 1670 cm^−^^1^; ^13^C (150 MHz, CDCl_3_) and ^1^H (600 MHz, CDCl_3_) NMR data, see Table 1 and Table 2; ESIMS: *m/z* 461 [M + Na]^+^, 463 [M + 2 + Na]^+^; HRESIMS: *m/z* 461.13377 (calculated for C_22_H_27_^35^ClO_7_ + Na, 461.13375).

Briarenol X (**2**): amorphous powder; [α]${}_{D}^{24}$ −14 (*c* 0.05, CHCl_3_); IR (ATR) ν_max_ 3484, 1777, 1729 cm^−1^; ^13^C (150 MHz, CDCl_3_) and ^1^H (600 MHz, CDCl_3_) NMR data, see Table 1 and Table 2; ESIMS: *m/z* 551 [M + Na]^+^, 553 [M + 2 + Na]^+^; HRESIMS: *m/z* 551.20198 (calculated for C_26_H_37_^35^ClO_9_ + Na, 551.20183).

Briarenol Y (**3**): amorphous powder; [α]${}_{D}^{24}$ −73 (*c* 0.1, CHCl_3_); IR (KBr) ν_max_ 3459, 1778, 1738 cm^−1^; ^13^C (100 MHz, CDCl_3_) and ^1^H (400 MHz, CDCl_3_) NMR data, see Table 1 and Table 2; ESIMS: *m/z* 565 [M + Na]^+^, 567 [M + 2 + Na]^+^; HRESIMS: *m/z* 565.18106 (calculated for C_26_H_35_^35^ClO_10_ + Na, 565.18110).

Briarenol Z (**4**): amorphous powder; [α]${}_{D}^{24}$ −12 (*c* 0.2, CHCl_3_); IR (KBr) ν_max_ 3450, 1777, 1736 cm^−1^; ^13^C (100 MHz, CDCl_3_) and ^1^H (400 MHz, CDCl_3_) NMR data, see Table 1 and Table 2; ESIMS: *m/z* 565 [M + Na]^+^, 567 [M + 2 + Na]^+^; HRESIMS: *m/z* 565.18137 (calculated for C_26_H_35_^35^ClO_10_ + Na, 565.18110).

Solenolide A (**5**): amorphous powder; [α]${}_{D}^{25}$ −28 (*c* 0.04, CHCl_3_) (reference [15], [α]${}_{D}^{20}$ −56 (*c* 0.63, CHCl_3_)); the ^1^H and ^13^C NMR data of **5** are in full agreement with those reported previously [15]; ESIMS: *m/z* 579 [M + Na]^+^, 581 [M + 2 + Na]^+^.

Briarenolide M (**6**): amorphous powder; [α]${}_{D}^{24}$ −81 (*c* 0.3, CHCl_3_) (reference [16], [α]${}_{D}^{25}$ −58 (*c* 0.7, CHCl_3_)); the ^1^H and ^13^C NMR data of **6** are in full agreement with those reported previously [16]; ESIMS: *m/z* 537 [M + Na]^+^, 539 [M + 2 + Na]^+^.

Briaexcavatolide F (**7**): amorphous powder; [α]${}_{D}^{26}$ −14 (*c* 0.1, MeOH) (reference [17], [α]${}_{D}^{25}$ −21 (*c* 0.1, MeOH)); the ^1^H and ^13^C NMR data of **7** are in full agreement with those reported previously [17]; ESIMS: *m/z* 565 [M + Na]^+^, 567 [M + 2 + Na]^+^.

Brianolide (**8**): colorless prisms; [α]${}_{D}^{26}$ −25 (*c* 2.3, CHCl_3_) (reference [18], [α]${}_{D}^{23}$ −15 (*c* 0.1, MeOH)); the ^1^H and ^13^C NMR data of **8** are in full agreement with those reported previously [18]; ESIMS: *m/z* 537 [M + Na]^+^, 539 [M + 2 + Na]^+^.

### 3.4. Single-Crystal X-Ray Crystallography of Brianolide (***8***)

Suitable colorless prisms of **1** were obtained from a solution of MeOH. The crystal (0.158 × 0.108 × 0.108 mm^3^) belongs to the tetragonal system, space group *P*4_1_2_1_2 (#92), with *a* = 10.9478(4) Å, *b* = 10.9478(4) Å, *c* = 46.0277(15) Å, *V* = 5516.6(4) Å^3^, *Z* = 8, *D*_calcd_ = 1.317 Mg/m^3^, *λ* (Cu Kα) = 1.54178 Å. Intensity data were measured on a Bruker D8 Venture diffractometer up to *θ*_max_ of 75.0°. All 24,967 reflections were collected. The structure was solved by direct methods and refined by a full-matrix least-squares procedure [27,28]. The refined structural model converged to a final R1 = 0.0578; wR2 = 0.1667 for 5687 observed reflections (*I* > 2*σ*(*I*)) and 348 variable parameters. The absolute configuration was determined by the Flack parameter x = 0.095(8) [29,30]. Crystallographic data for the structure of brianolide (**8**) were deposited with the Cambridge Crystallographic Data Center (CCDC) under supplementary publication number CCDC 1966097 [31].

### 3.5. Molecular Mechanics Calculations

The MM2 force field [19] in CHEM3D PRO software from CambridgeSoft Corporation (version 15.0, Cambridge, MA, USA) was used to calculate the molecular models.

### 3.6. In Vitro Inflammatory Assay

The inflammatory assay was employed to evaluate the activities of briaranes **1**‒**7** related to the release of iNOS and COX-2 from macrophage cells, as reported in the literature [32].

## 4. Conclusions

Eight chlorinated briarane diterpenoids, including four new briaranes—briarenols W‒Z (**1**‒**4**)—as well as four known analogues—solenolide A (**5**), briarenolide M (**6**), briaexcavatolide F (**7**), and brianolide (**8**)—were identified from a cultured octocoral *B. stechei*, originally flourishing in Taiwanese waters where the Kuroshio current and South China Sea surface current converge to provide high biodiversity. The structures of new briaranes **1**‒**4** were elucidated on the basis of spectroscopic analysis and the absolute configuration of **8** (brianolide) was determined by a single-crystal x-ray diffraction analysis. As briaranes **1**‒**7** were isolated along with brianolide (**8**) from the same target organism, *B. stechei*, it is reasonable on biogenetic grounds to assume that **1**‒**7** have the same absolute configuration as that of **8**, while the protons H-10 and Me-15 at C-1 in briaranes **1**‒**8** are α- and β-oriented, respectively, and these compounds have 1*S*,10*S*- configurations in the ring junction. Briaranes **2** (briarenol X) and **5** (solenolide A) displayed enhancing effects on the production of iNOS and COX-2 at a concentration of 10 μM.

## Figures and Tables

**Table 1:** ^13^C NMR data for briaranes **1**–**4**.

Position	1 ^a^	2 ^a^	3 ^b^	4 ^b^
1	44.2, C ^c^	40.4, C	39.1, C	38.4, C
2	76.3, CH	82.0, CH	76.2, CH	74.1, CH
3	35.4, CH_2_	25.3, CH_2_	60.5, CH	62.4, CH
4	76.6, CH	21.2, CH_2_	58.1, CH	56.1, CH
5	137.5, C	140.3, C	137.9, C	137.4, C
6	54.9, CH	64.9, CH	126.1, CH	63.2, CH
7	80.4, CH	76.3, CH	76.7, CH	83.0, CH
8	82.4, C	86.1, C	81.7, C	80.9, C
9	76.9, CH	71.8, CH	69.1, CH	69.5, CH
10	45.8, CH	33.9, CH	36.5, CH	35.9, CH
11	42.1, CH	36.8, CH	36.5, CH	36.0, CH
12	205.6, C	70.3, CH	71.6, CH	72.2, CH
13	126.0, CH	49.7, CH	57.2, CH	57.2, CH
14	156.3, CH	62.1, CH	62.1, CH	62.0, CH
15	16.2, CH_3_	20.2, CH_3_	15.2, CH_3_	15.3, CH_3_
16	116.1, CH_2_	118.6, CH_2_	44.1, CH_2_	121.2 CH_2_
17	50.2, CH	44.4, CH	43.6, CH	46.1, CH
18	8.2, CH_3_	7.2, CH_3_	6.3, CH_3_	6.0, CH_3_
19	175.3, C	176.4, C	175.5, C	173.8, C
20	15.4, CH_3_	14.5, CH_3_	9.7, CH_3_	9.4, CH_3_
OAc-2	170.6, C	168.3, C		
	21.1, CH_3_	20.9, CH_3_		
OAc-9			169.5, C	169.9, C
			21.8, CH_3_	21.8, CH_3_
*n*-butyrate-12		173.3, C	173.1, C	173.1, C
		35.8, CH_2_	36.2, CH_2_	36.2, CH_2_
		18.4, CH_2_	18.4, CH_2_	18.4, CH_2_
		13.7, CH_3_	13.6, CH_3_	13.6, CH_3_

^a^ 150 MHz, CDCl_3_, ^b^ 100 MHz, CDCl_3_, ^c^ Multiplicity deduced by ^13^C, distortionless enhancement by polarization transfer (DEPT), and heteronuclear single quantum coherence (HSQC) spectra.

**Table 2:** ^1^H NMR data (*J* in Hz) for briaranes **1**–**4**.

Position	1 ^a^	2 ^a^	3 ^b^	4 ^b^
2	4.86 d (7.2)	5.17 d (7.2)	3.11 dd (9.6, 2.8)	3.58 dd (8.8, 4.0)
3α	1.46 dd (15.6, 4.2)	1.66 m		
β	3.32 ddd (15.6, 13.2, 7.2)	2.41 m	3.38 dd (9.2, 4.0)	3.33 dd (8.8, 4.4)
4α		2.08 m		
β	4.87 dd (13.2, 4.2)	2.01 m	4.14 dd (4.0, 2.0)	3.91 d (4.4)
6	5.52 m	5.02 br s	6.03 ddd (9.2, 1.2, 1.2)	4.92 d (10.4)
7	4.65 d (3.0)	5.35 d (1.8)	5.28 d (9.2)	4.71 d (10.4)
9	4.53 d (6.0)	3.57 dd (7.2, 7.2)	5.29 d (8.0)	5.33 d (8.4)
10	2.08 d (12.0)	2.63 br s	1.77 dd (8.0, 2.8)	1.82 dd (8.4, 2.4)
11	2.72 dq (12.0, 7.2)	2.15 m	2.14 m	1.95 m
12		4.74 dd (5.4, 1.8)	4.71 d (4.8)	4.58 d (4.8)
13	5.95 d (10.8)	3.47 dd (5.4, 3.6)	3.19 dd (3.6, 0.8)	3.24 d (4.0)
14	6.95 d (10.8)	2.88 d (3.6)	3.29 d (3.6)	3.27 d (4.0)
15	1.39 s	1.30 s	1.19 s	1.16 s
16a/b	5.34 d (1.8); 5.48 d (1.8)	5.72 s; 5.89 s	4.19 d (12.4); 4.09 d (12.4)	5.40 s; 5.64 s
17	2.53 q (7.2)	3.11 q (7.2)	2.42 q (7.2)	2.76 q (7.2)
18	1.22 d (7.2)	1.14 d (7.2)	1.19 d (7.2)	1.18 d (7.2)
20	1.30 d (7.2)	1.05 d (7.8)	1.03 d (7.2)	1.01 d (7.2)
OH-2			2.43 s	2.35 d (4.0)
OH-8		2.86 br s	3.12 s	3.06 s
OH-9	2.84 d (6.0)			
OAc-2	2.15 s	2.19 s		
OAc-9			2.22 s	2.23 s
*n*-butyrate-12		2.34 t (7.2)	2.35 t (7.2)	2.35 t (7.2)
		1.69 sext (7.2)	1.67 sext (7.2)	1.67 sext (7.2)
		0.99 t (7.2)	0.96 t (7.2)	0.96 t (7.2)

^a^ 600 MHz, CDCl_3_, ^b^ 400 MHz, CDCl_3_.

**Table 3:** Effects of briaranes **1**–**7** on lipopolysaccharide (LPS)-induced pro-inflammatory inducible nitric oxide synthase (iNOS) and cyclooxygenase-2 (COX-2) protein expressions in macrophages.

Compound	iNOS	COX-2	*β*-Actin
10 µM	Expression (% of LPS)		
Control	2.88 ± 0.86	0.94 ± 0.10	107.01 ± 2.73
Vehicle	100.00 ± 1.84	100.00 ± 3.98	100.00 ± 1.66
1	88.18 ± 12.38	103.23 ± 5.20	101.63 ± 4.23
2	142.03 ± 18.44	159.21 ± 13.41	97.81 ± 3.15
3	99.71 ± 13.77	89.20 ± 1.40	98.31 ± 5.33
4	103.25 ± 16.72	96.92 ± 4.72	99.46 ± 3.75
5	134.11 ± 14.70	196.03 ± 12.35	106.56 ± 1.98
6	86.20 ± 11.20	85.98 ± 2.47	104.50 ± 2.01
7	92.55 ± 10.52	91.71 ± 1.90	104.80 ± 2.53
Dexamethasone	61.24 ± 11.09	18.17 ± 2.65	104.70 ± 3.83

Data were normalized to those of cells treated with LPS alone and cells treated with dexamethasone were used as a positive control. Data are expressed as the mean ± SEM. The β-actin of Western blotting was used for loading/internal control.

## Data Availability

Any data not included in the manuscript or supplementary materials that supports the work presented in this manuscript is available upon reasonable request to the corresponding author (P.-J.S.).

## Supplementary Materials

The following are available online at https://www.mdpi.com/1660-3397/19/2/77/s1, ESIMS, HRESIMS, IR, 1D (^1^H and ^13^C) and 2D (HSQC, HMBC, COSY, and NOESY) NMR spectra of briarenols W–Z (**1**–**4**).
