# Supplementary material for: Briarenols W–Z: Chlorine-Containing Polyoxygenated Briaranes from Octocoral Briareum stechei (Kükenthal, 1908)
Source: Mar Drugs. 2021 Jan 31;19(2):77. doi: 10.3390/md19020077 (PMC7911717; doi:10.3390/md19020077)
Supplement: Supplementary file 1 [file marinedrugs-19-00077-s001.pdf]

S1. ESIMS spectrum of compound **1** 3
S2. HRESIMS spectrum of compound **1** 4
S3. IR spectrum of compound **1** 4
S4. ^{1}H NMR spectrum (600 MHz) of compound **1** in $CDCl_3$ 5
S5. Zoomed-in region of Figure S4 from 2.0-3.4 ppm 6
S6. Zoomed-in region of Figure S4 from 4.4-7.0 ppm 7
S7. ^{13}C NMR spectrum (150 MHz) of compound **1** in $CDCl_3$ 8
S8. HSQC spectrum of compound **1** in $CDCl_3$ 9
S9. HMBC spectrum of compound **1** in $CDCl_3$ 10
S10. ^{1}H-^{1}H COSY spectrum of compound **1** in $CDCl_3$ 11
S11. NOESY spectrum of compound **1** in $CDCl_3$ 12
S12. ESIMS spectrum of compound **2** 13
S13. HRESIMS spectrum of compound **2** 14
S14. IR spectrum of compound **2** 14
S15. ^{1}H NMR spectrum (600 MHz) of compound **2** in $CDCl_3$ 15
S16. Zoomed-in region of Figure S15 from 0.8-3.2 ppm 16
S17. Zoomed-in region of Figure S15 from 3.4-6.0 ppm 17
S18. ^{13}C NMR spectrum (150 MHz) of compound **2** in $CDCl_3$ 18
S19. HSQC spectrum of compound **2** in $CDCl_3$ 19
S20. HMBC spectrum of compound **2** in $CDCl_3$ 20
S21. ^{1}H-^{1}H COSY spectrum of compound **2** in $CDCl_3$ 21
S22. NOESY spectrum of compound **2** in $CDCl_3$ 22
S23. ESIMS spectrum of compound **3** 23
S24. HRESIMS spectrum of compound **3** 24
S25. IR spectrum of compound **3** 24
S26. ^{1}H NMR spectrum (400 MHz) of compound **3** in $CDCl_3$ 25
S27. Zoomed-in region of Figure S26 from 0.8-2.5 ppm 26
S28. Zoomed-in region of Figure S26 from 3.0-6.1 ppm 27
S29. ^{13}C NMR spectrum (100 MHz) of compound **3** in $CDCl_3$ 28
S30. HSQC spectrum of compound **3** in $CDCl_3$ 29
S31. HMBC spectrum of compound **3** in $CDCl_3$ 30
S32. ^{1}H-^{1}H COSY spectrum of compound **3** in $CDCl_3$ 31
S33. NOESY spectrum of compound **3** in $CDCl_3$ 32
S34. ESIMS spectrum of compound **4** 33
S35. HRESIMS spectrum of compound **4** 34
S36. IR spectrum of compound **4** 34
S37. ^{1}H NMR spectrum (400 MHz) of compound **4** in $CDCl_3$ 35
S38. Zoomed-in region of Figure S37 from 0.9-2.5 ppm 36

S39. Zoomed-in region of Figure S37 from 2.7-5.9 ppm.. 37
S40. ^{13}C NMR spectrum (100 MHz) of compound **4** in $CDCl_3$ 38
S41. HSQC spectrum of compound **4** in $CDCl_3$... 39
S42. HMBC spectrum of compound **4** in $CDCl_3$.. 40
S43. ^{1}H-^{1}H COSY spectrum of compound **4** in $CDCl_3$.. 41
S44. NOESY spectrum of compound **4** in $CDCl_3$.. 42

FT-MS

Analysis Info

Analysis Name	D:\Data\a2\BP3265348_000014.d	10/15/2020 3:24:25 PM
Method	broadband first signal	
Sample Name	BP3-2-6-5-3-4-8	Instrument: FT-MS solariX
Comment	ESI Positive	

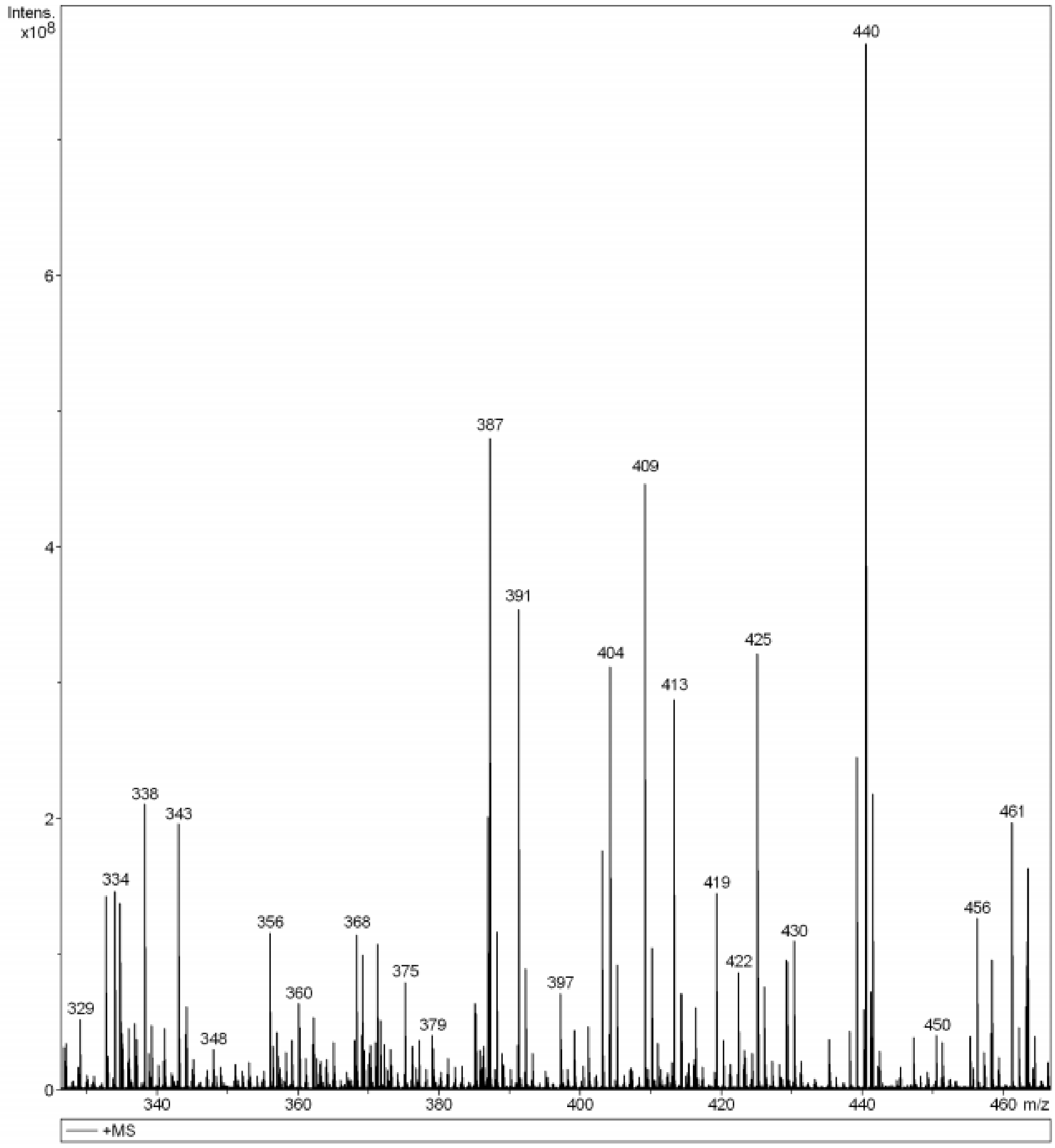

Bruker Compass DataAnalysis 4.0 printed: 10/15/2020 3:24:25 PM Page 1 of 1

S1. ESIMS spectrum of compound **1**

Mass Spectrum SmartFormula Report

Analysis Info

Analysis Name	D:\Data\a2\BP3265348_000013.d		10/15/2020 3:22:54 PM
Method	broadband first signal		Operator: YU HSIAO-CHING
Sample Name	BP3-2-6-5-3-4-8		Instrument: BRUKER FT-MS solariX
Comment	ESI Positive		

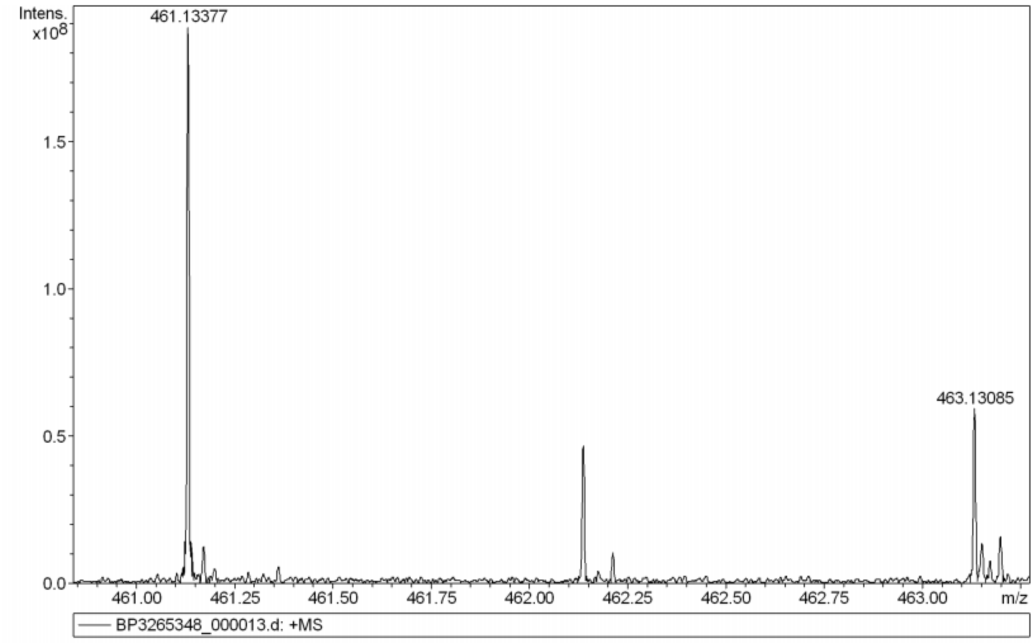

Meas. m/z	#	Formula	Score	m/z	err [mDa]	err [ppm]	mSigma	rdb	e⁻ Conf	N-Rule
461.13377	1	C 22 H 27 Cl Na O 7	100.00	461.13375	-0.02	-0.03	23.4	8.5	even	ok

S2. HRESIMS spectrum of compound **1**

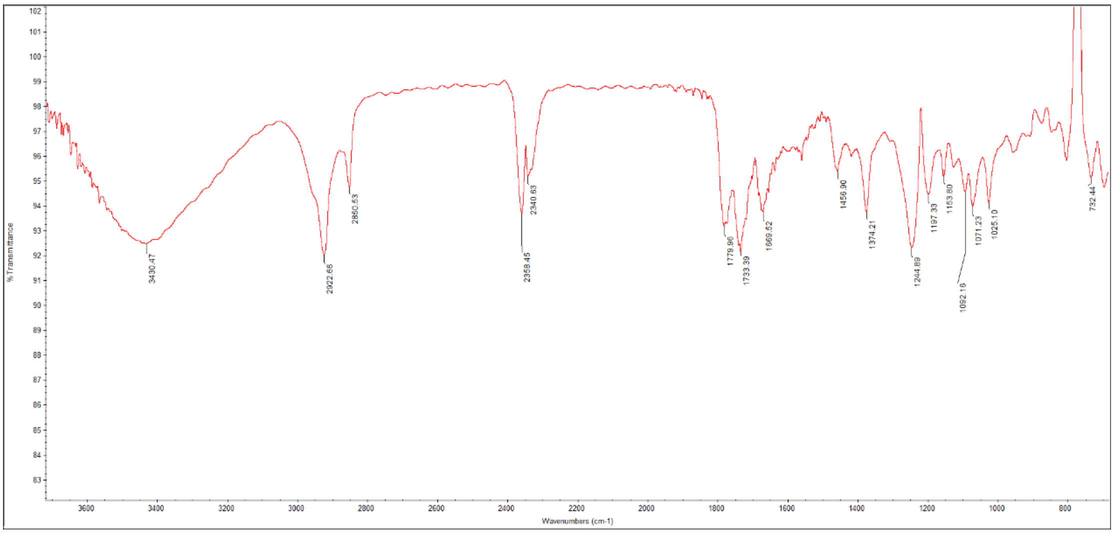

S3. IR spectrum of compound **1**

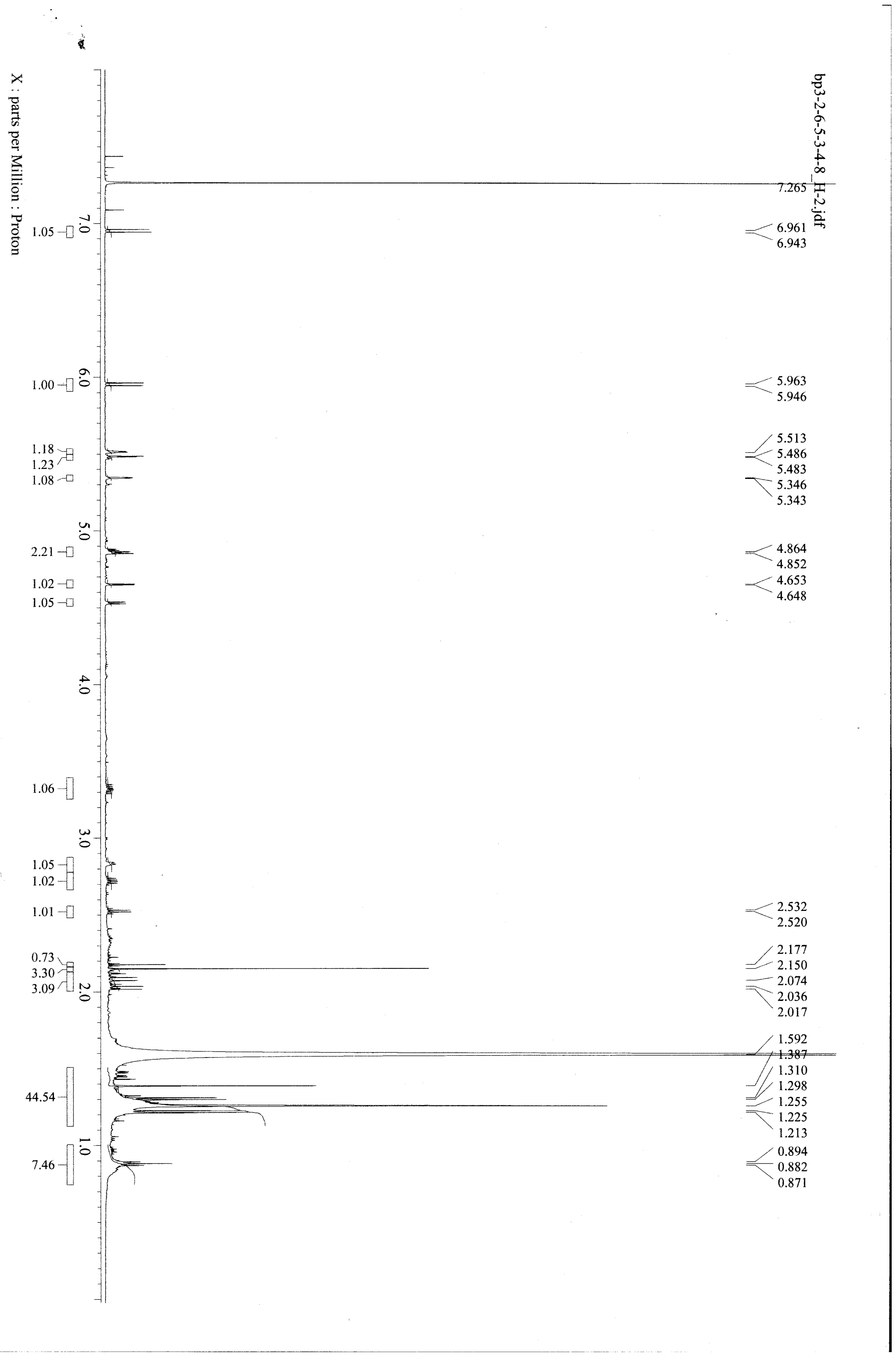

S4. ^{1}H NMR spectrum (600 MHz) of compound **1** in $CDCl_3$

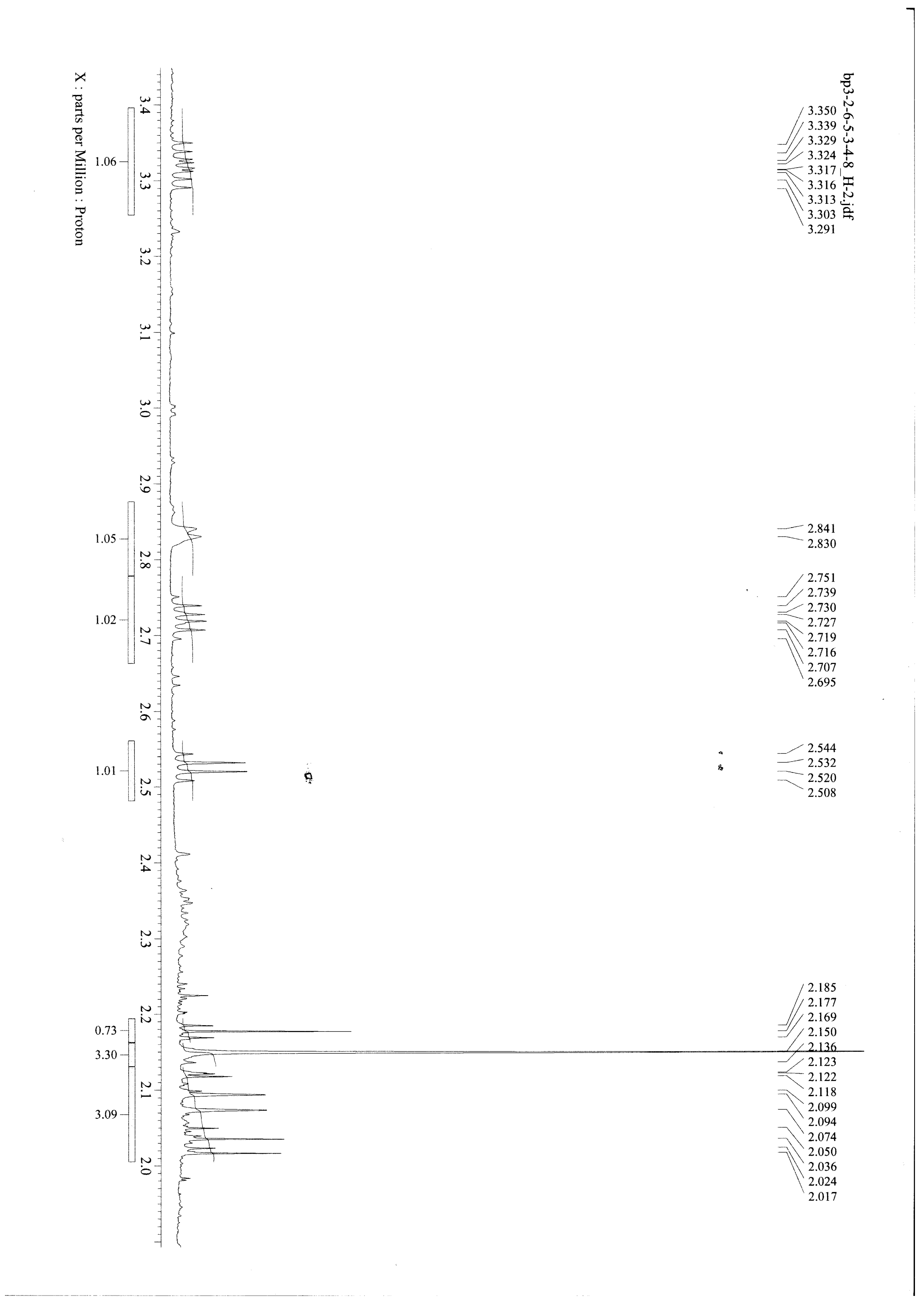

S5. Zoomed-in region of Figure S4 from 2.0-3.4 ppm

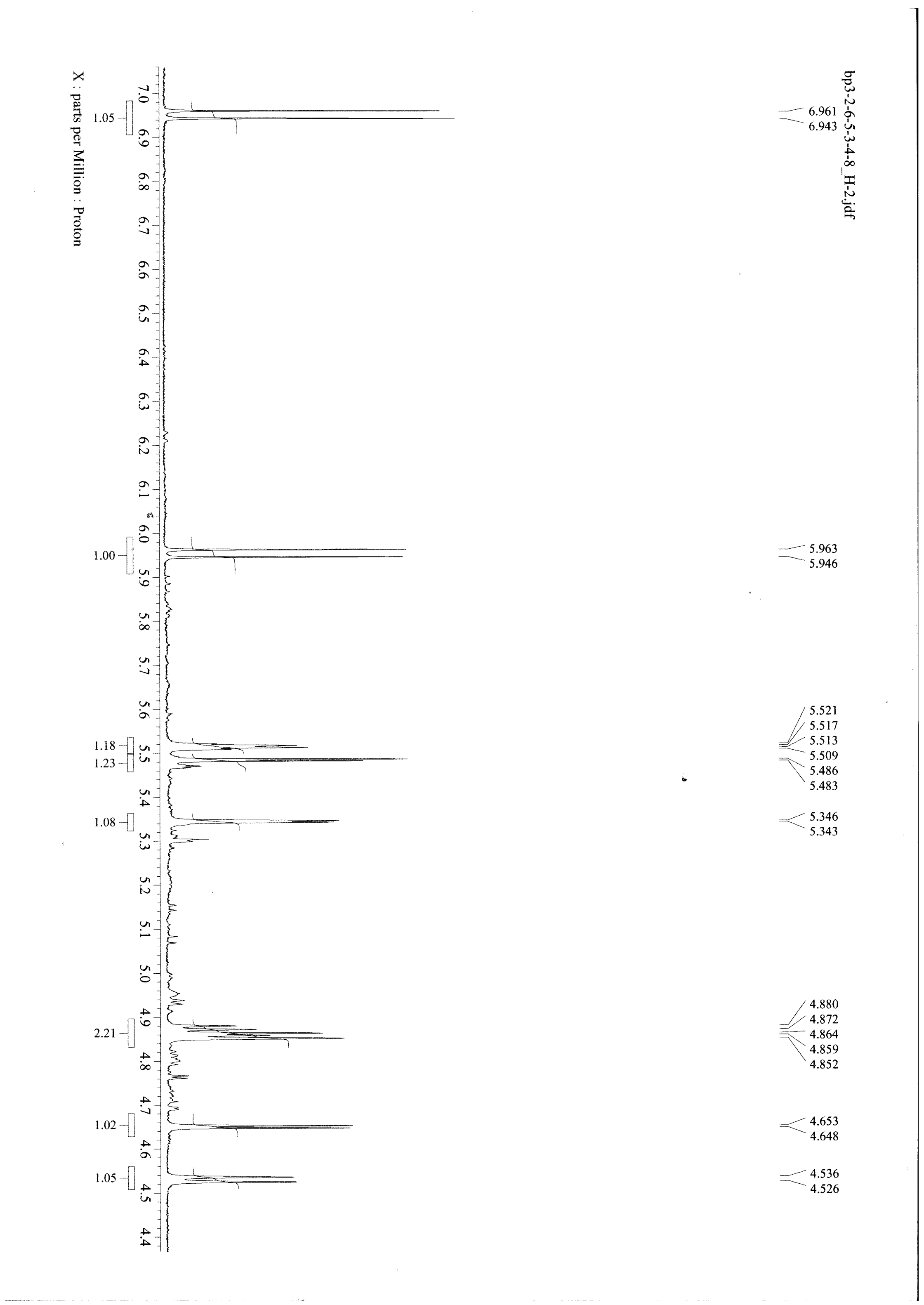

S6. Zoomed-in region of Figure S4 from 4.4-7.0 ppm

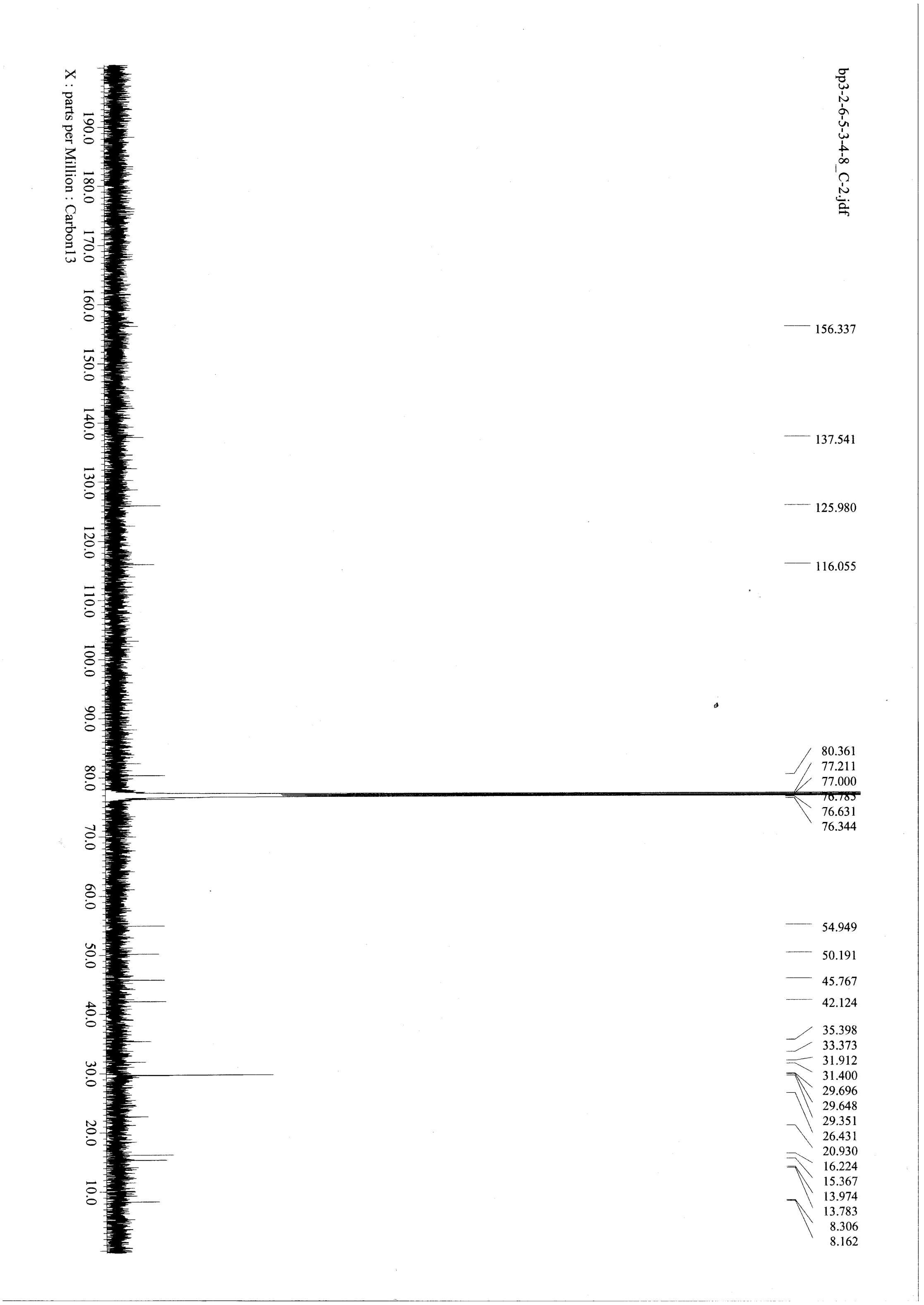

S7. ^{13}C NMR spectrum (150 MHz) of compound **1** in $CDCl_3$

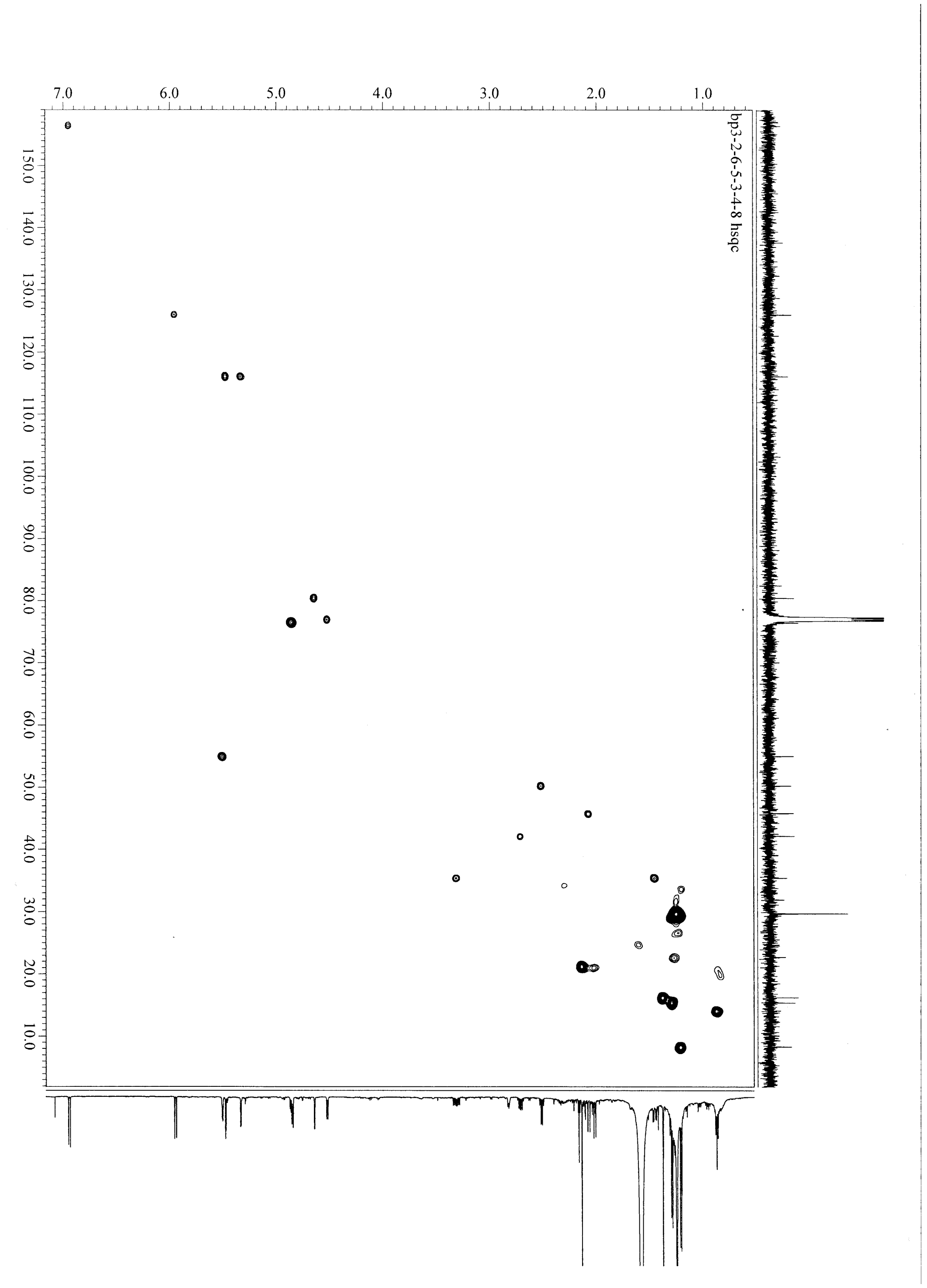

S8. HSQC spectrum of compound **1** in $CDCl_3$

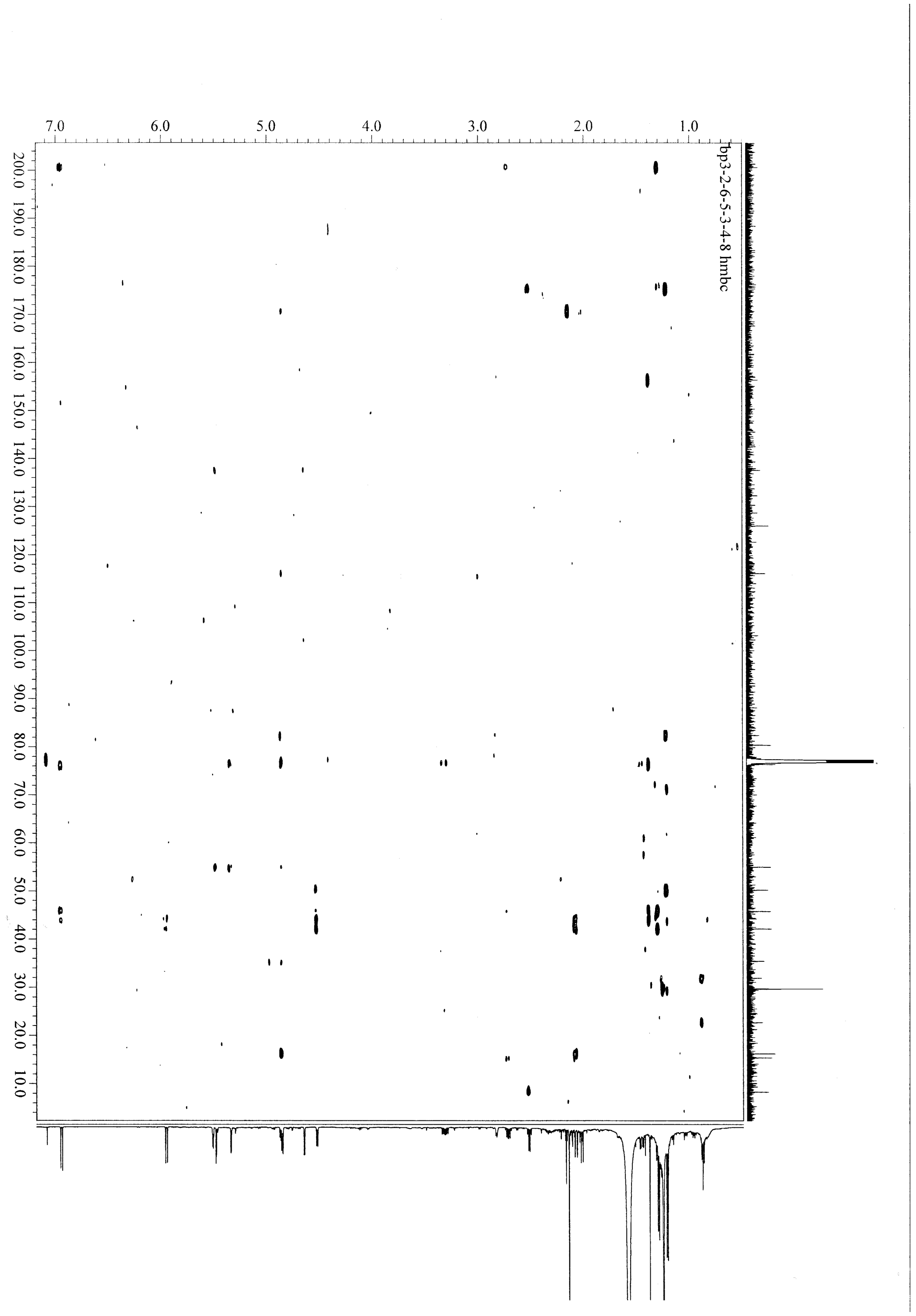

S9. HMBC spectrum of compound **1** in $CDCl_3$

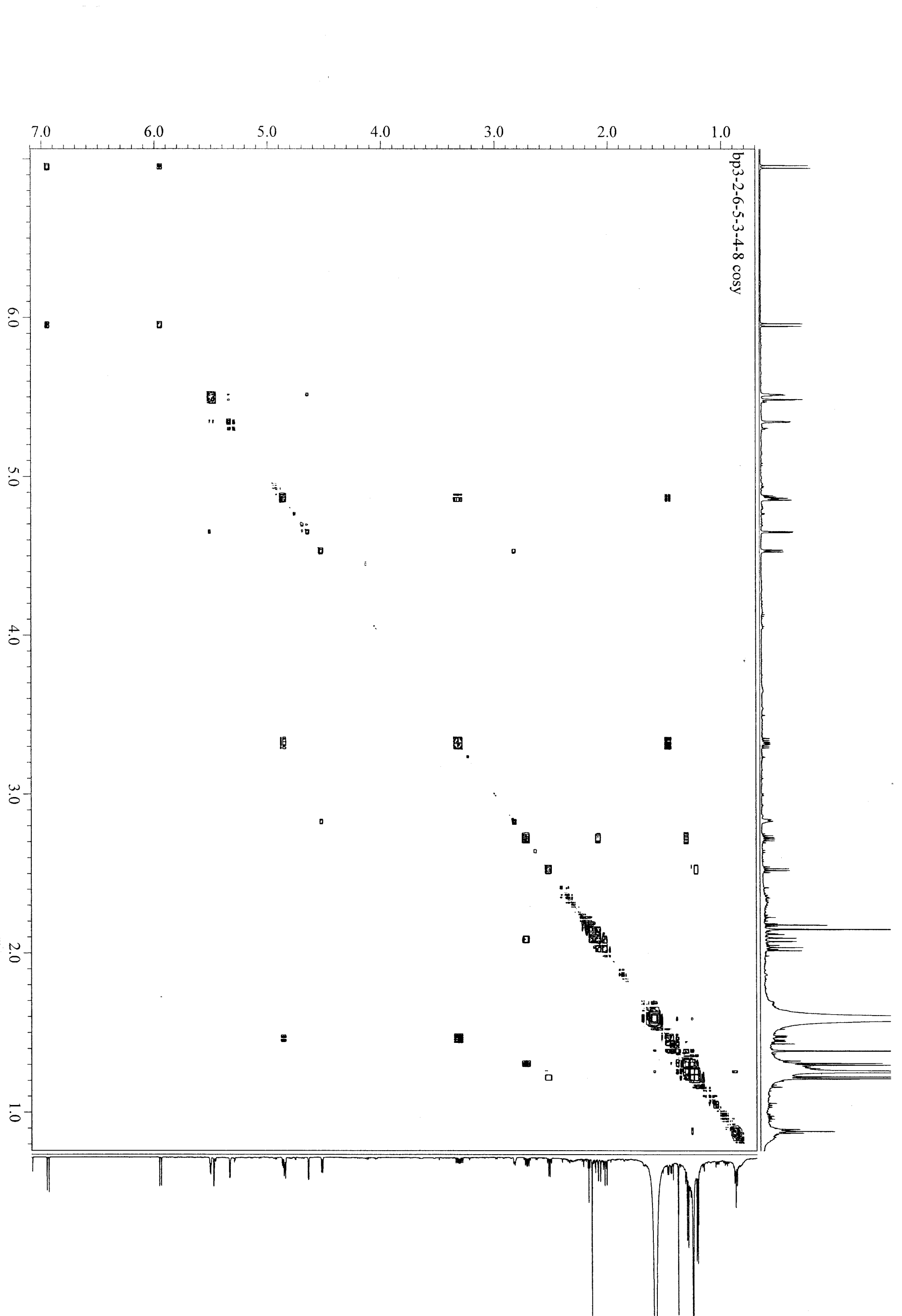

S10. ^{1}H-^{1}H COSY spectrum of compound **1** in $CDCl_3$

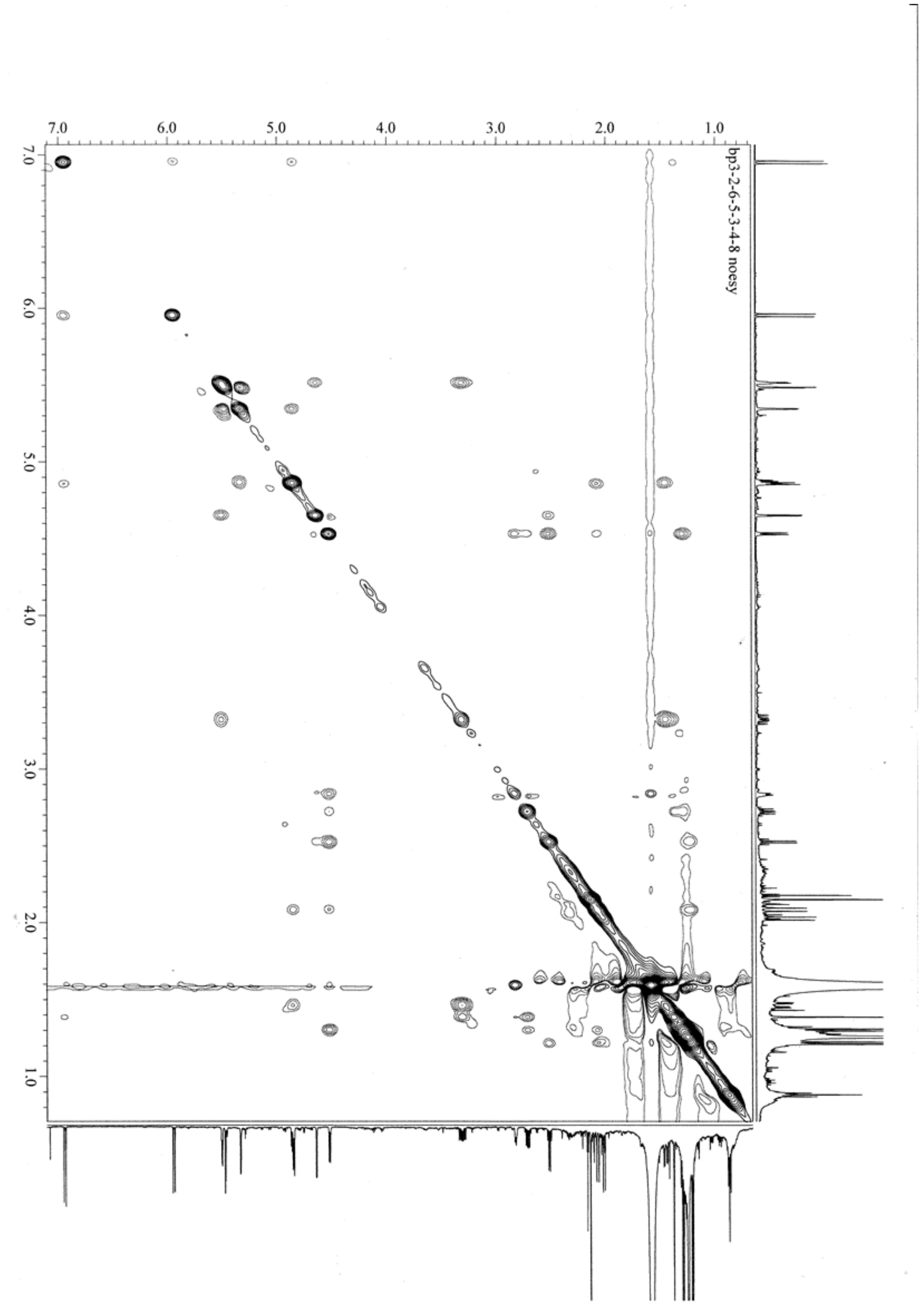

S11. NOESY spectrum of compound **1** in $CDCl_3$

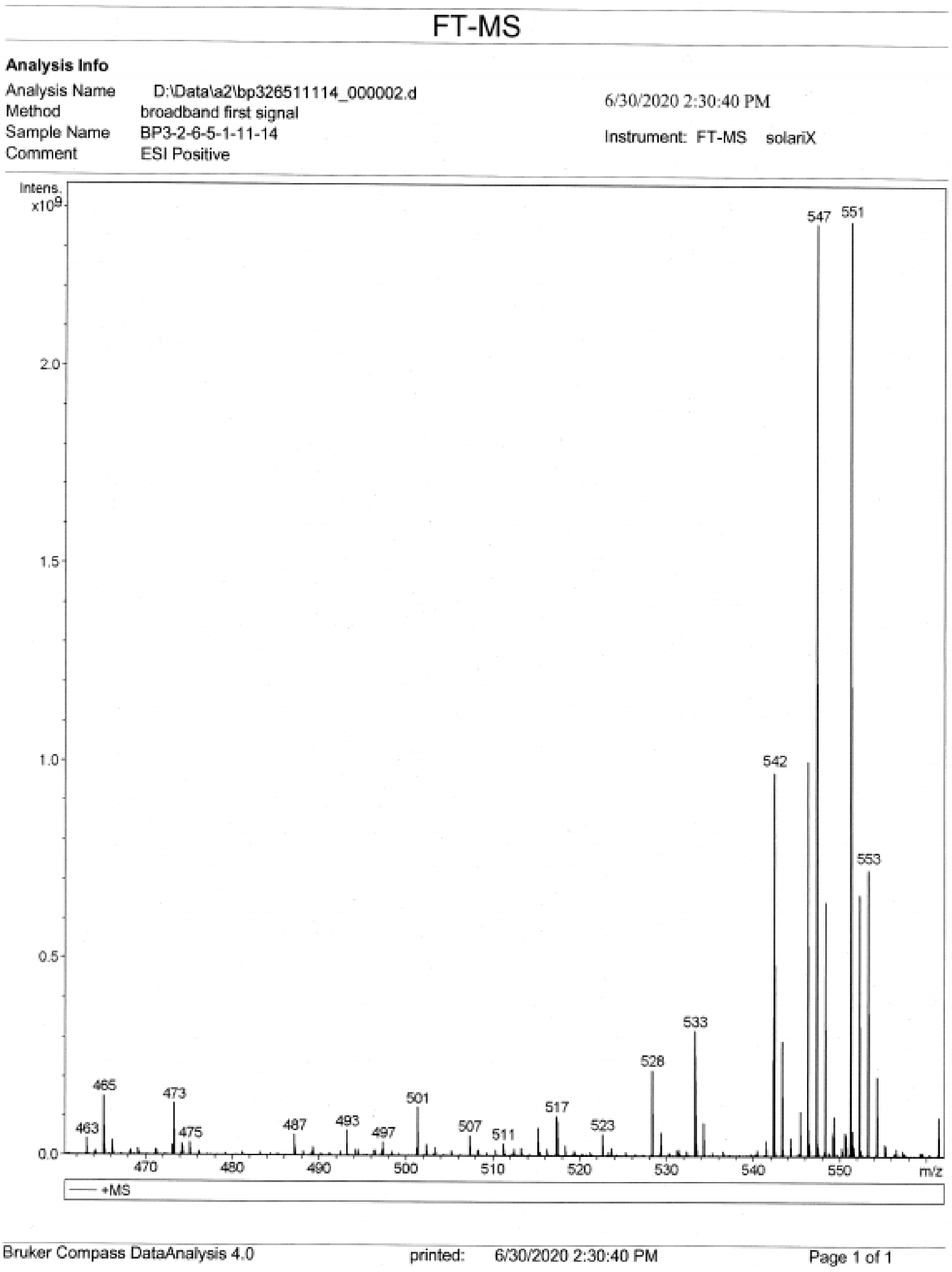

S12. ESIMS spectrum of compound **2**

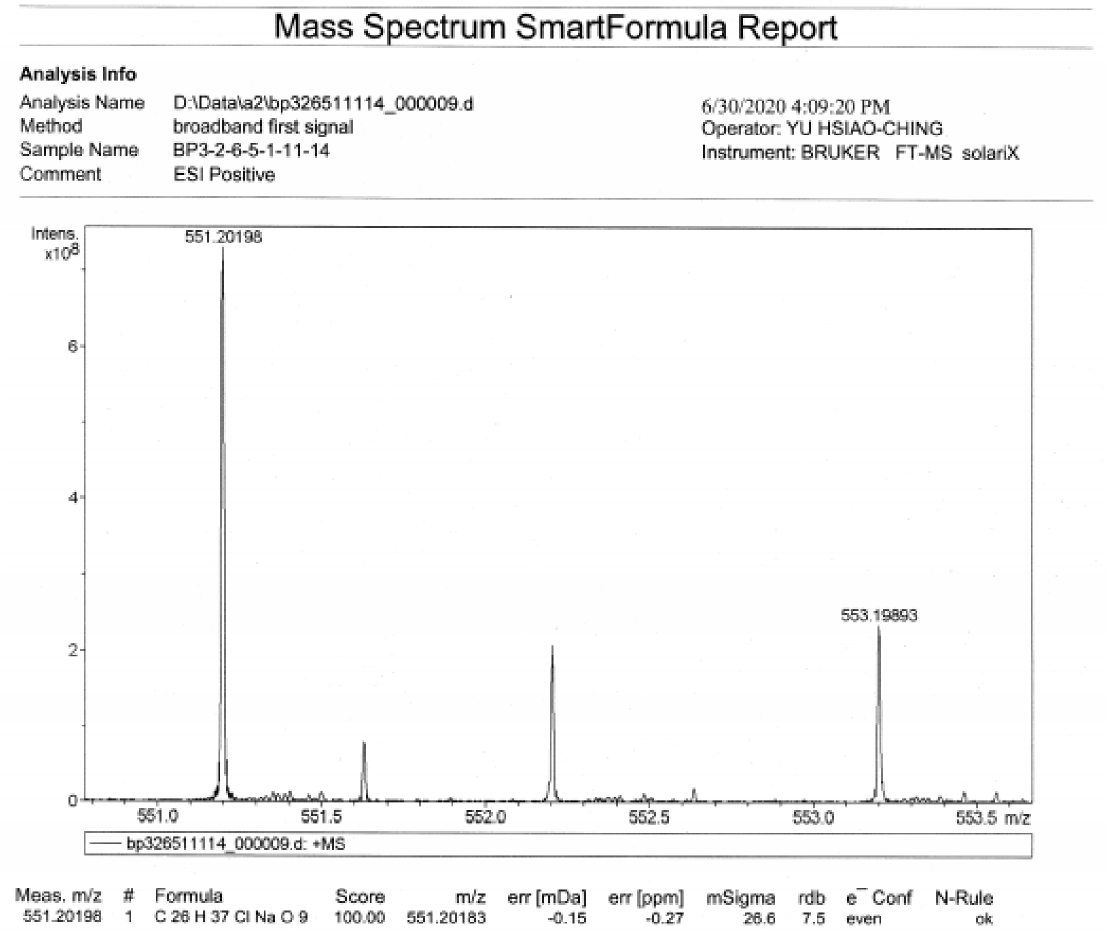

S13. HRESIMS spectrum of compound **2**

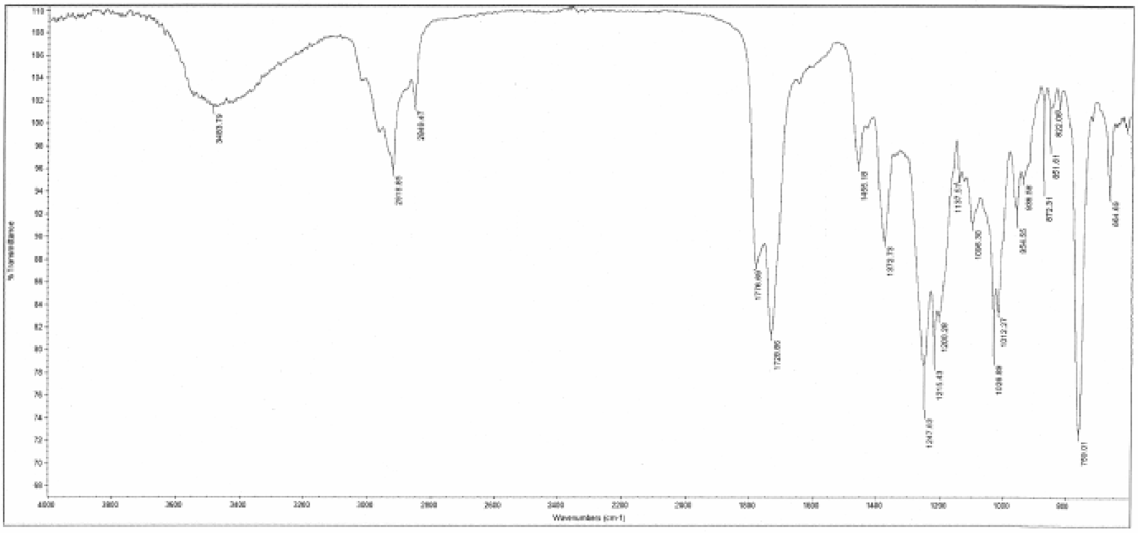

S14. IR spectrum of compound **2**

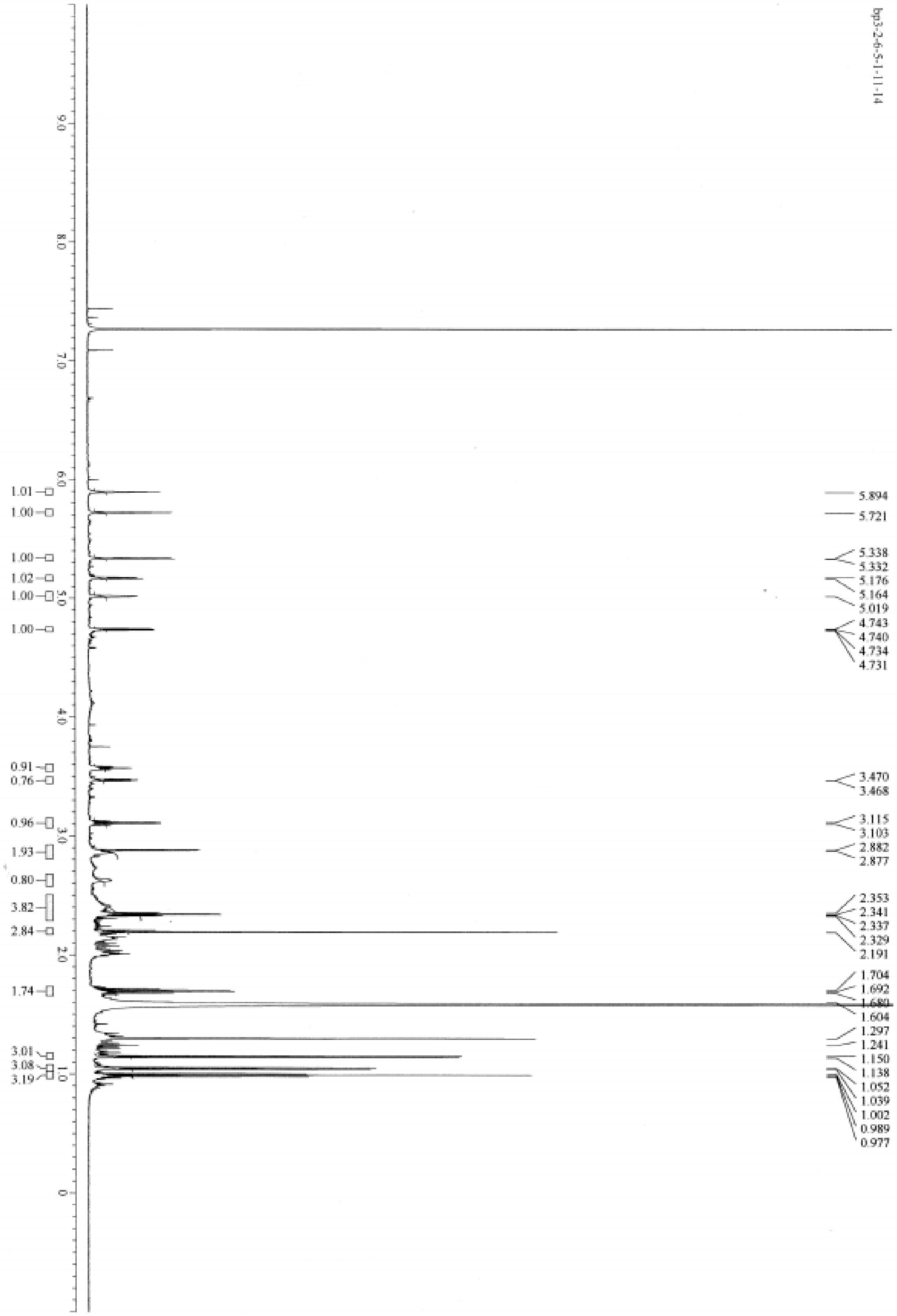

S15. ^{1}H NMR spectrum (600 MHz) of compound **2** in $CDCl_3$

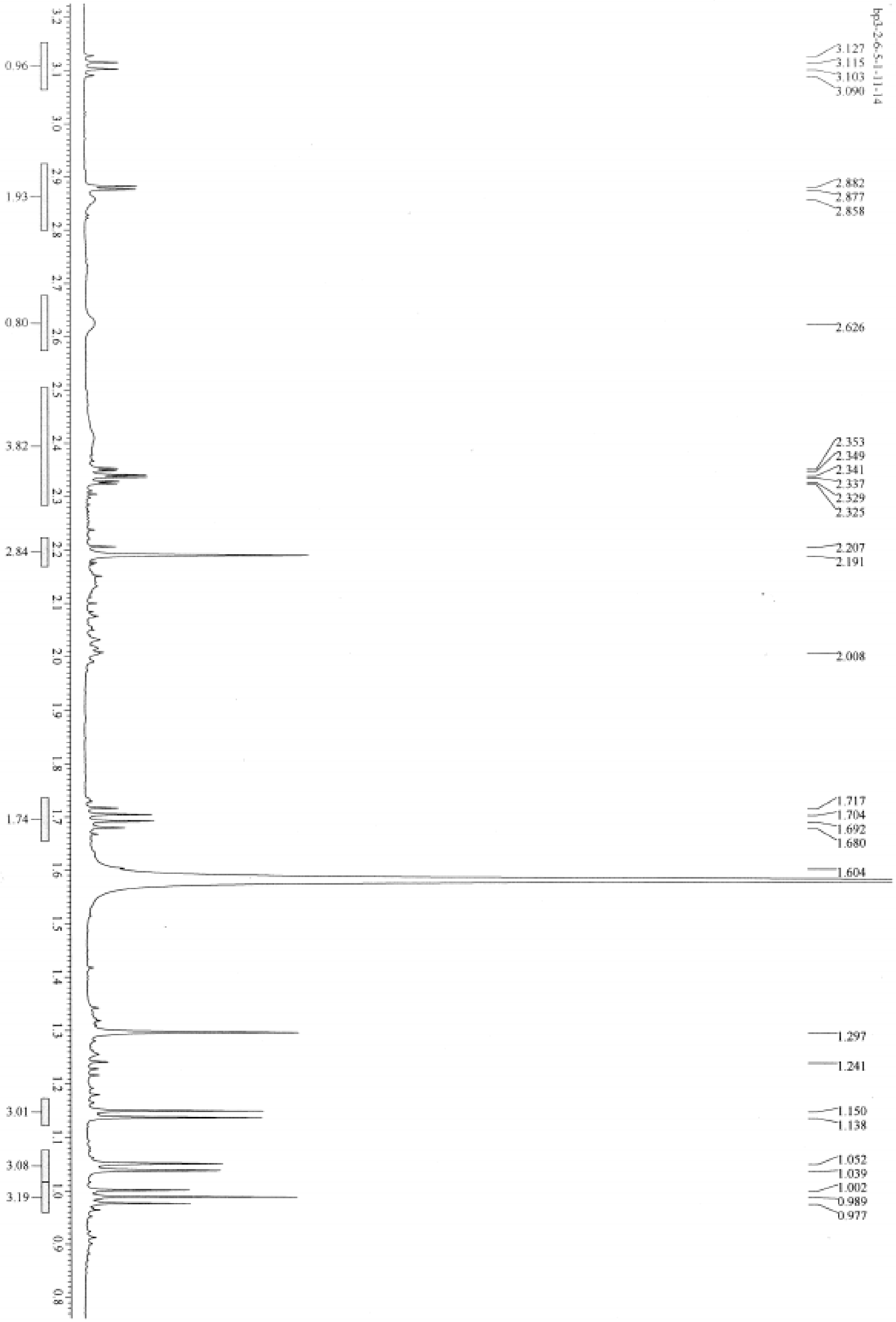

S16. Zoomed-in region of Figure S15 from 0.8-3.2 ppm

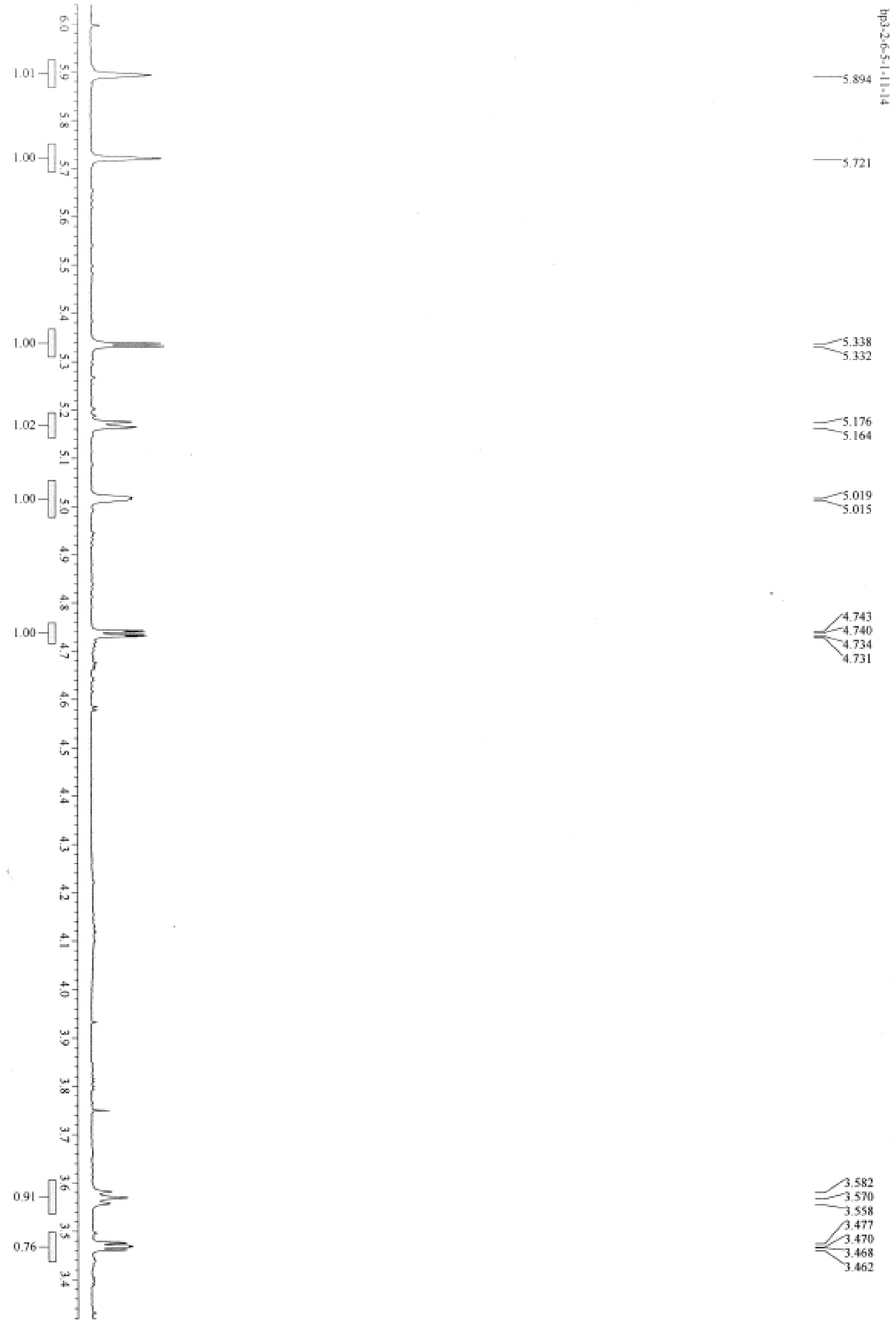

S17. Zoomed-in region of Figure S15 from 3.4-6.0 ppm

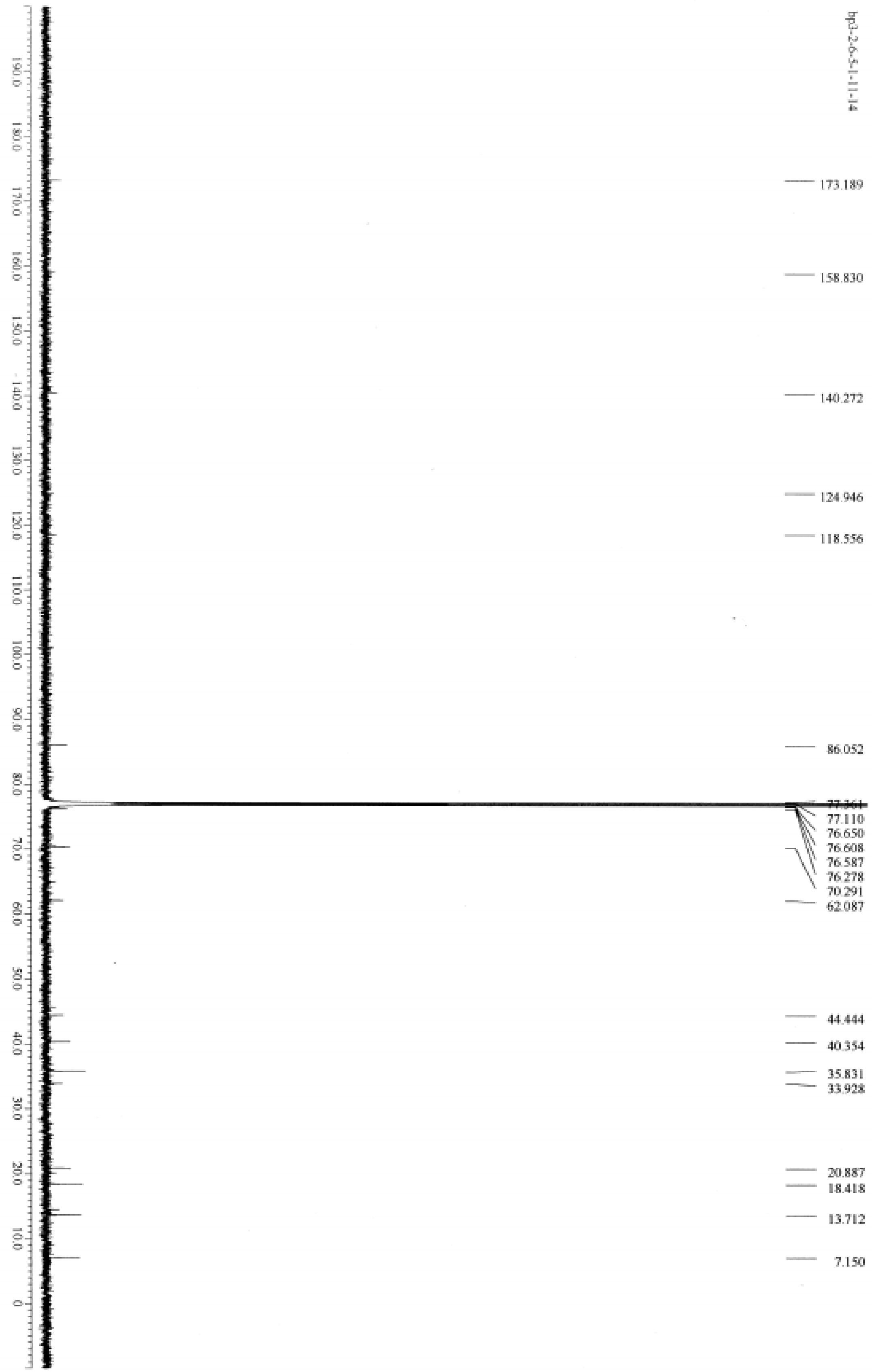

S18. ^{13}C NMR spectrum (150 MHz) of compound **2** in $CDCl_3$

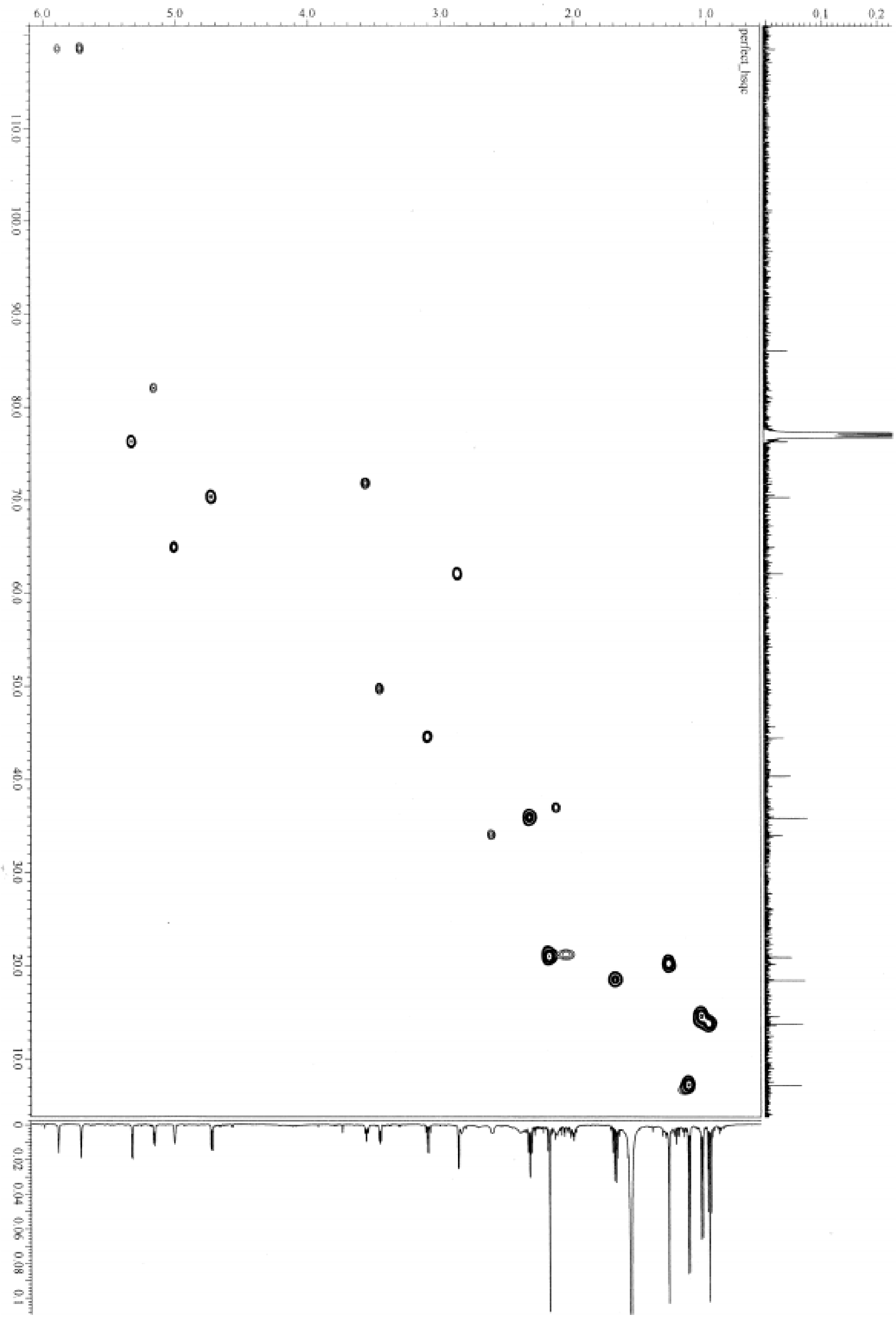

S19. HSQC spectrum of compound **2** in $CDCl_3$

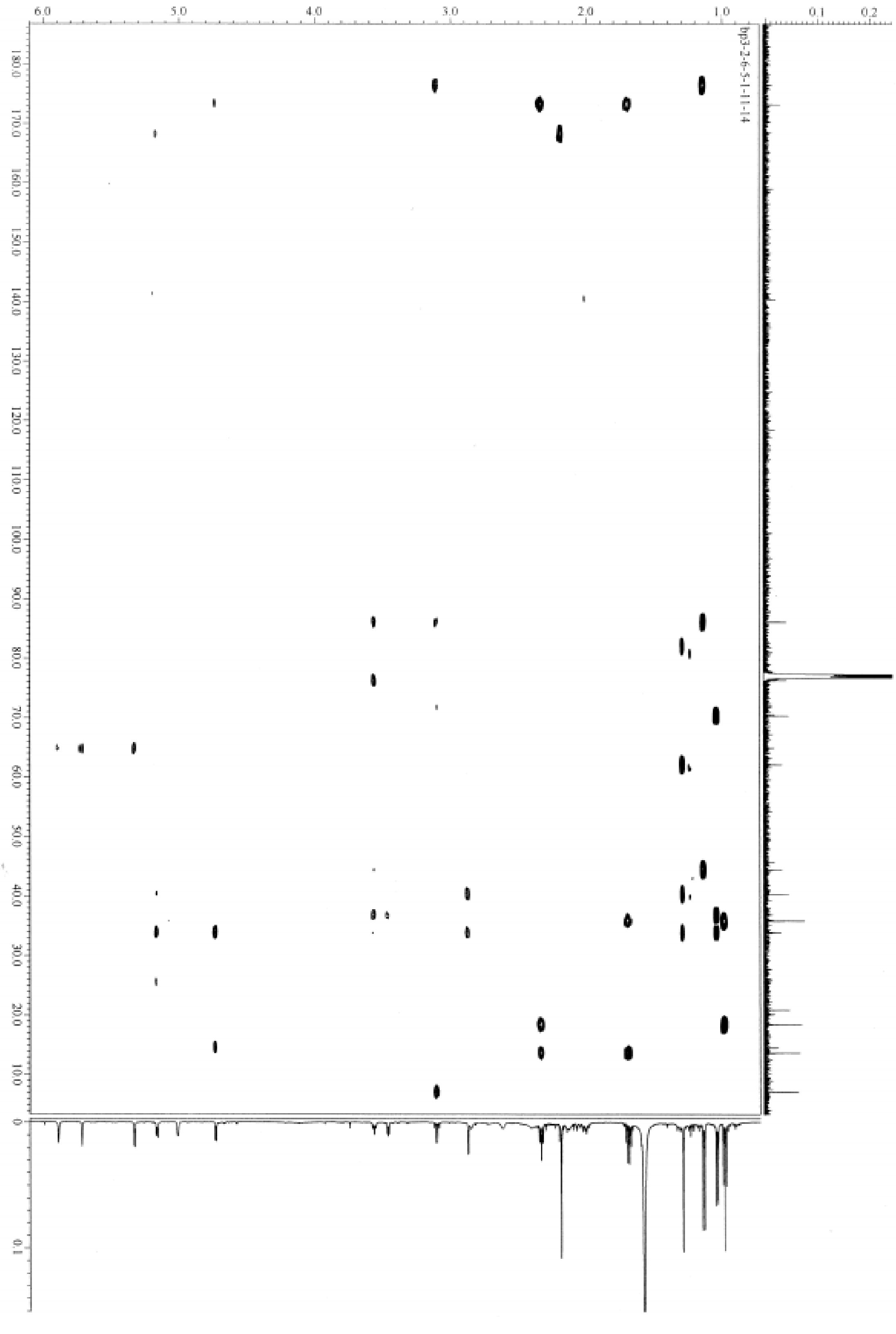

S20. HMBC spectrum of compound **2** in $CDCl_3$

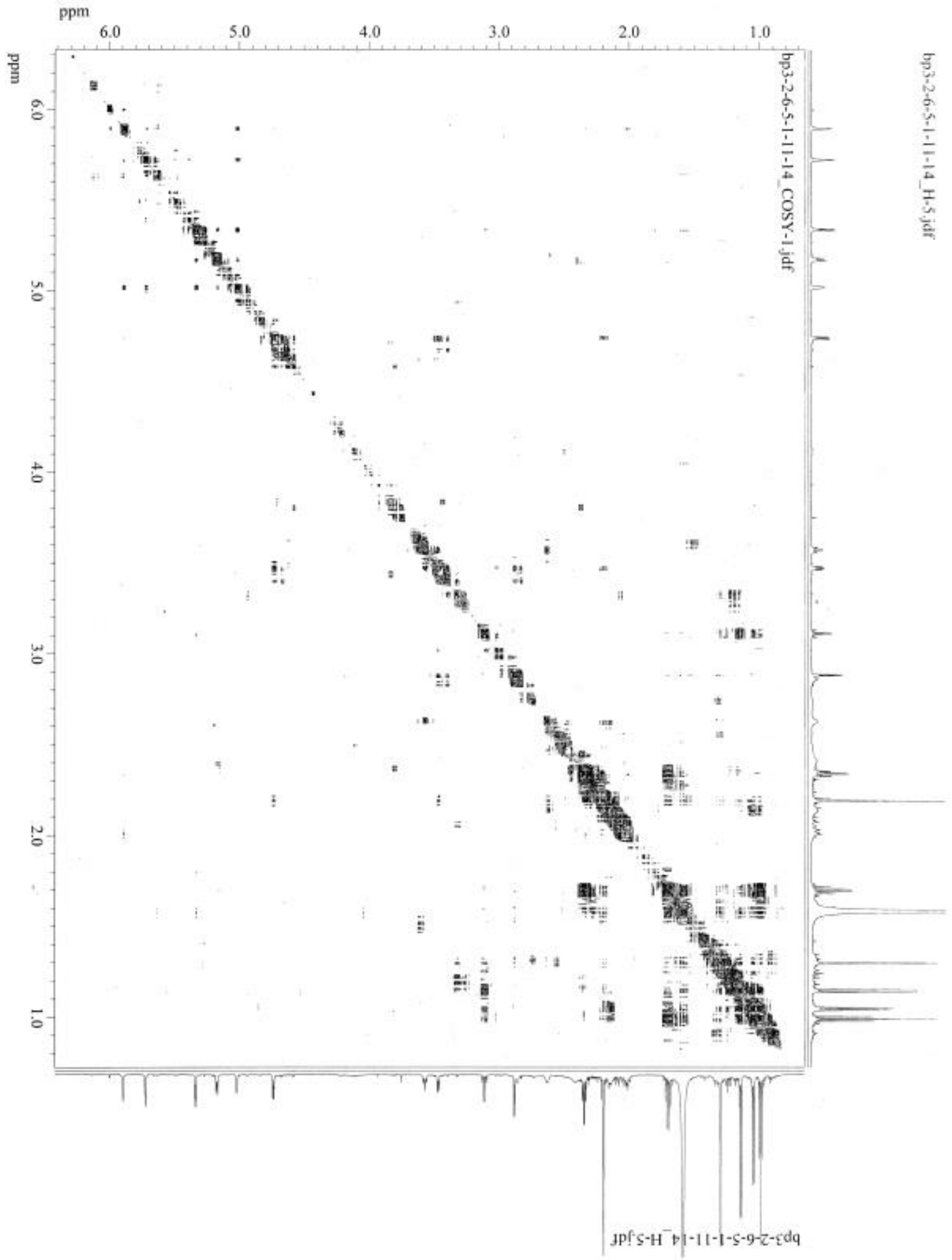

S21. ^{1}H-^{1}H COSY spectrum of compound **2** in $CDCl_3$

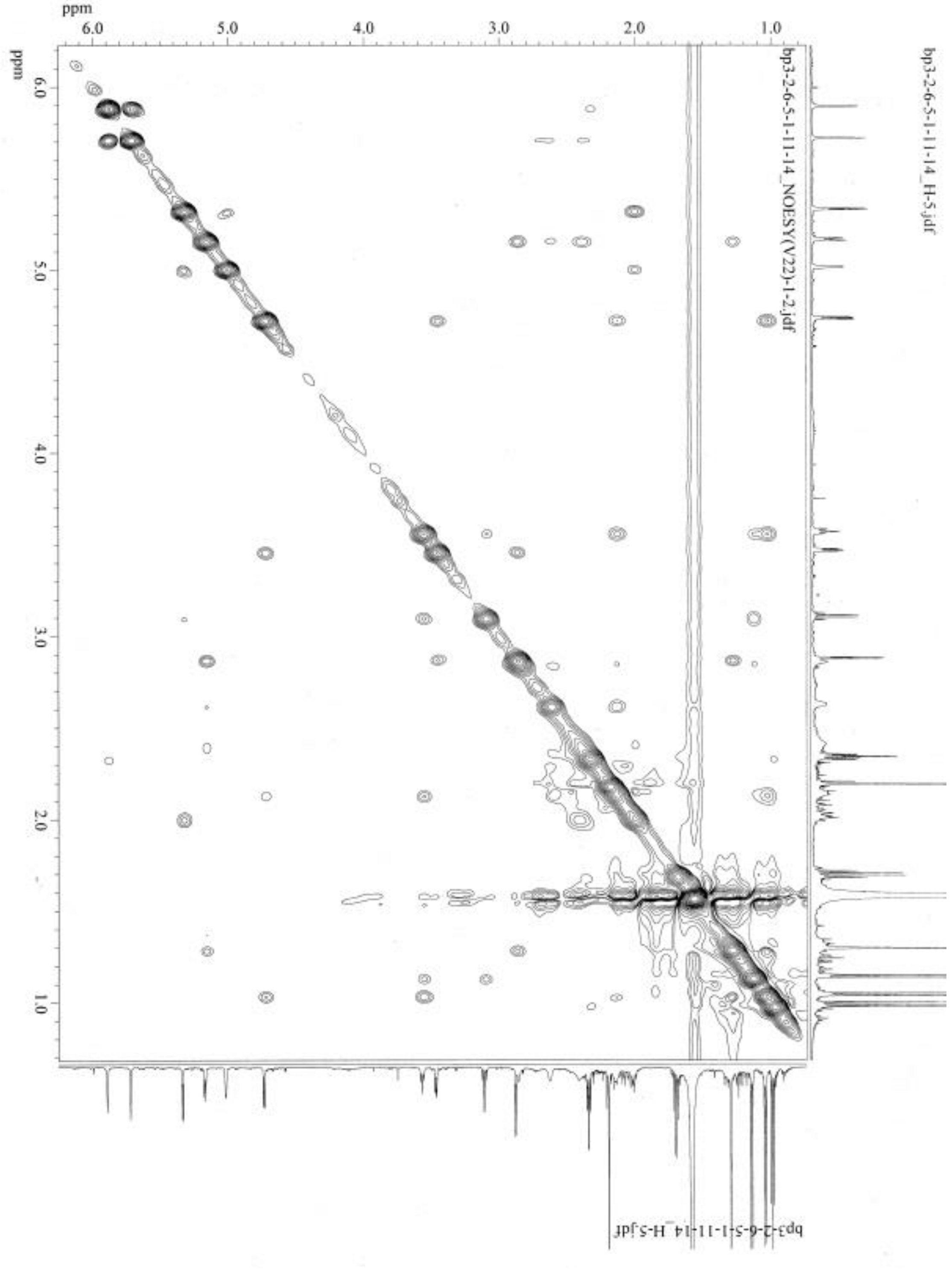

S22. NOESY spectrum of compound **2** in $CDCl_3$

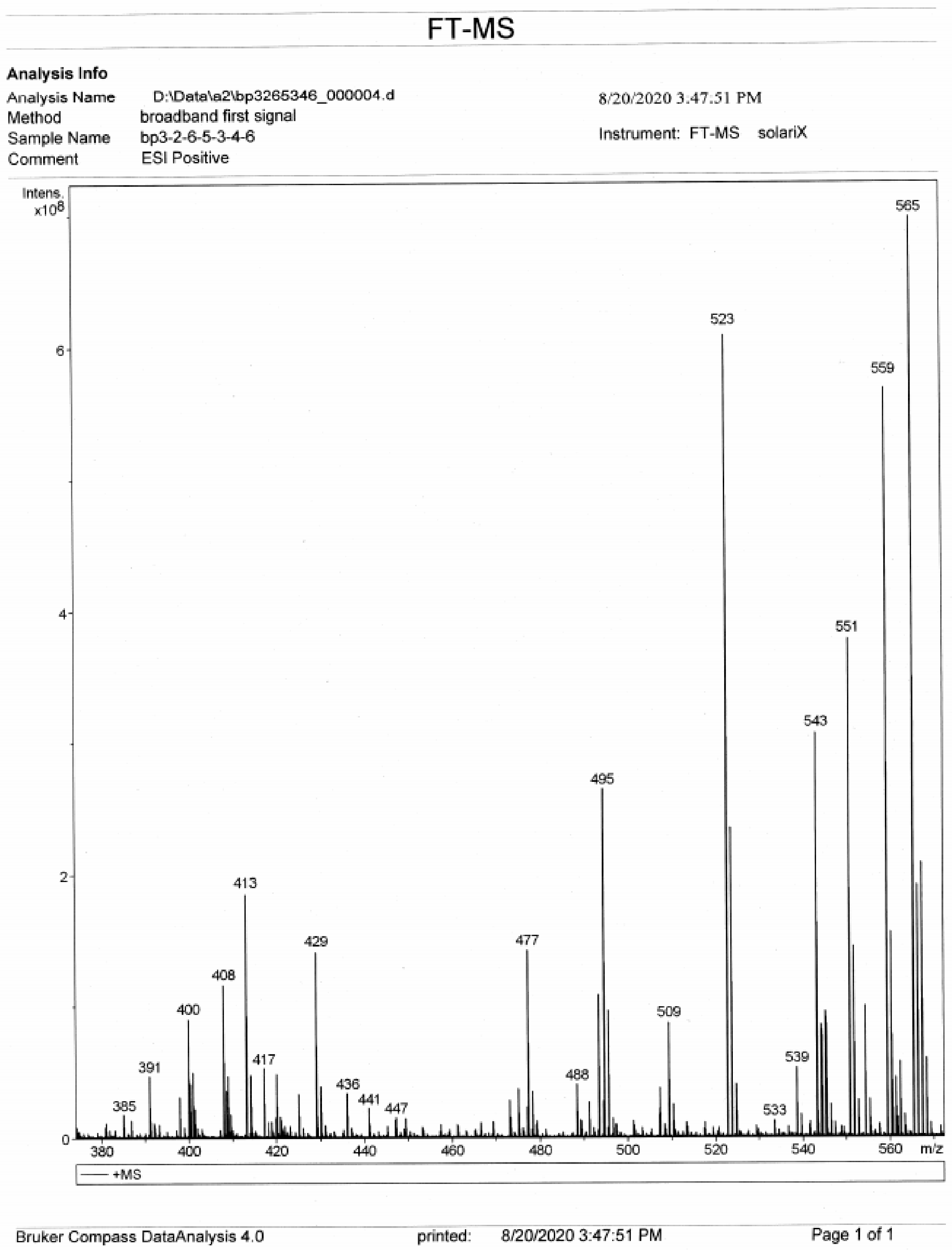

S23. ESIMS spectrum of compound **3**

Mass Spectrum SmartFormula Report

Analysis Info

Analysis Name	D:\Data\a2\bp3265346_000003.d	8/20/2020 3:46:31 PM
Method	broadband first signal	Operator: YU HSIAO-CHING
Sample Name	bp3-2-6-5-3-4-6	Instrument: BRUKER FT-MS solariX
Comment	ESI Positive	

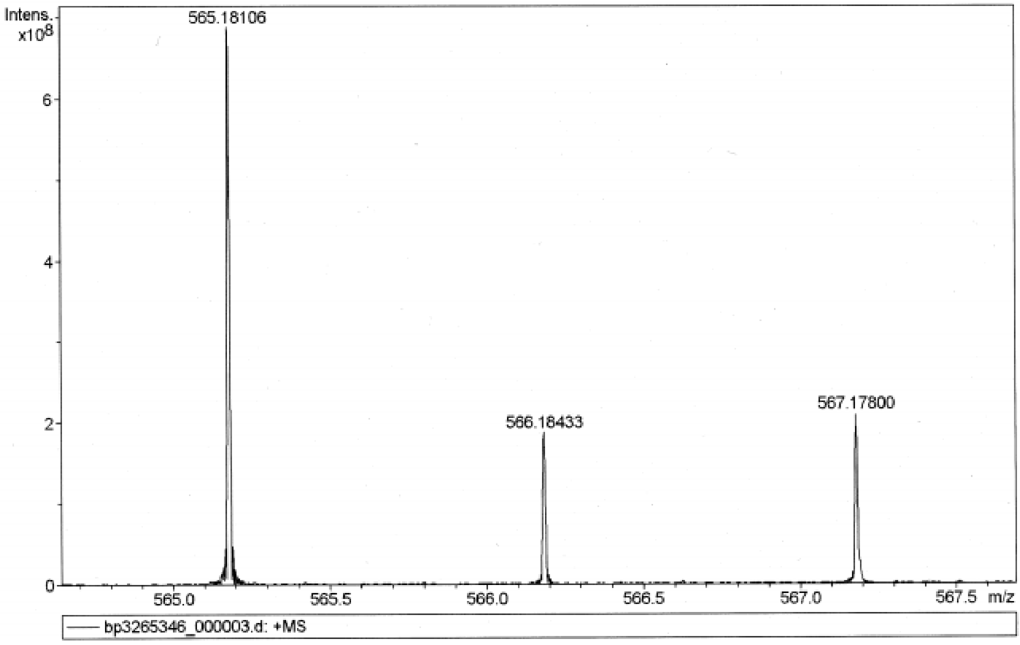

Meas. m/z	#	Formula	Score	m/z	err [mDa]	err [ppm]	mSigma	rdb	e⁻ Conf	N-Rule
565.18106	1	C 26 H 35 Cl Na O 10	100.00	565.18110	0.04	0.07	34.7	8.5	even	ok

S24. HRESIMS spectrum of compound **3**

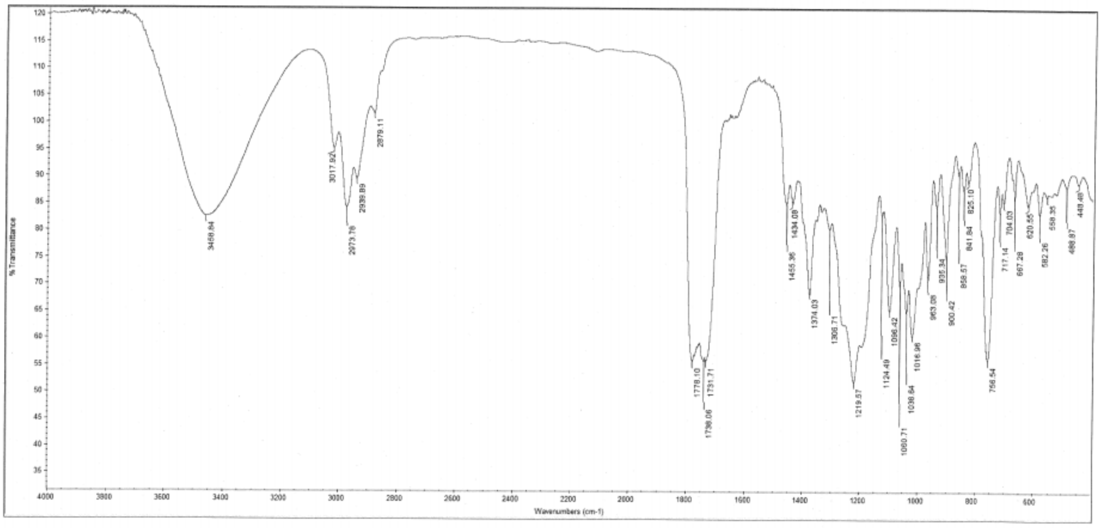

S25. IR spectrum of compound **3**

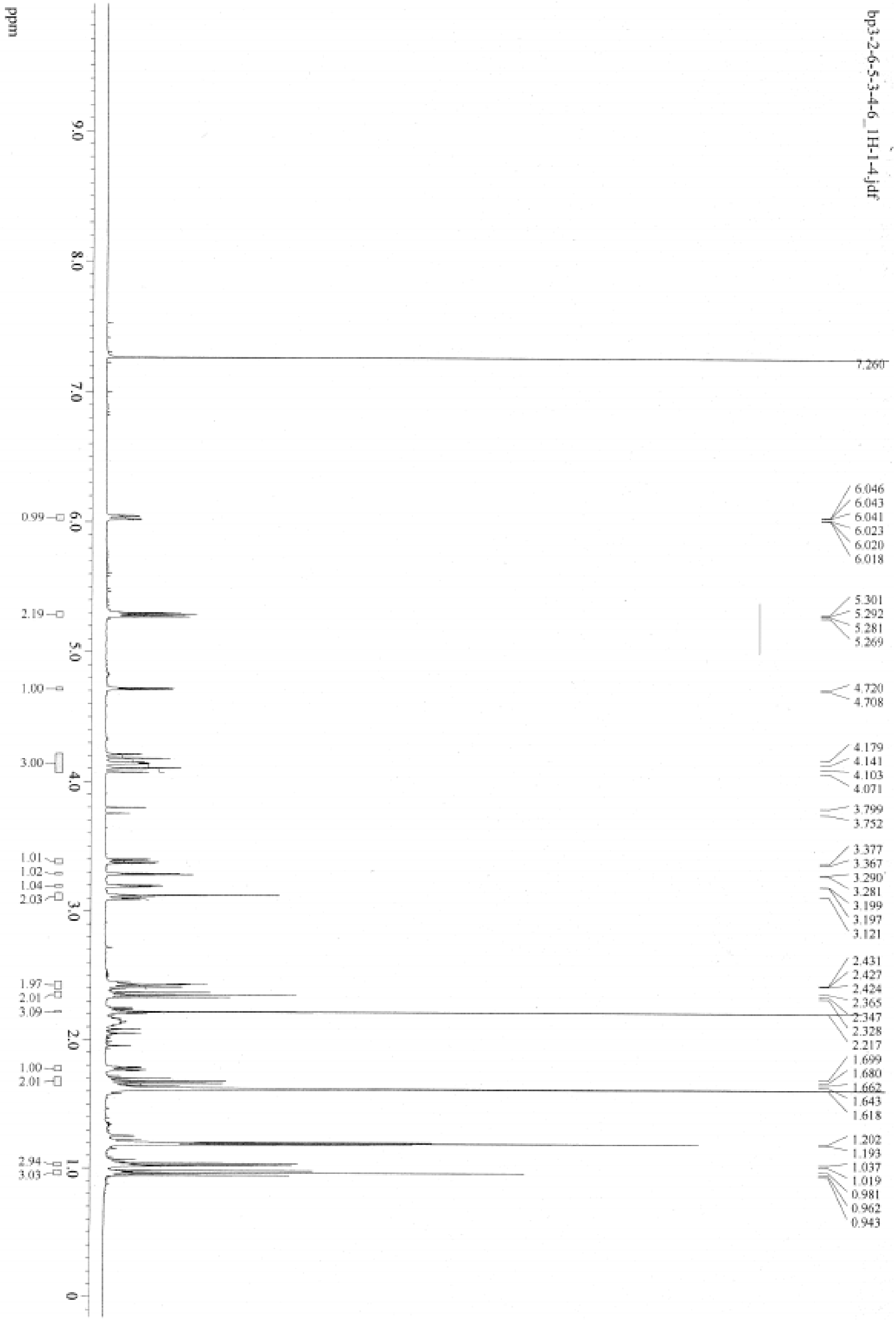

S26. ^1H NMR spectrum (600 MHz) of compound **3** in $CDCl_3$

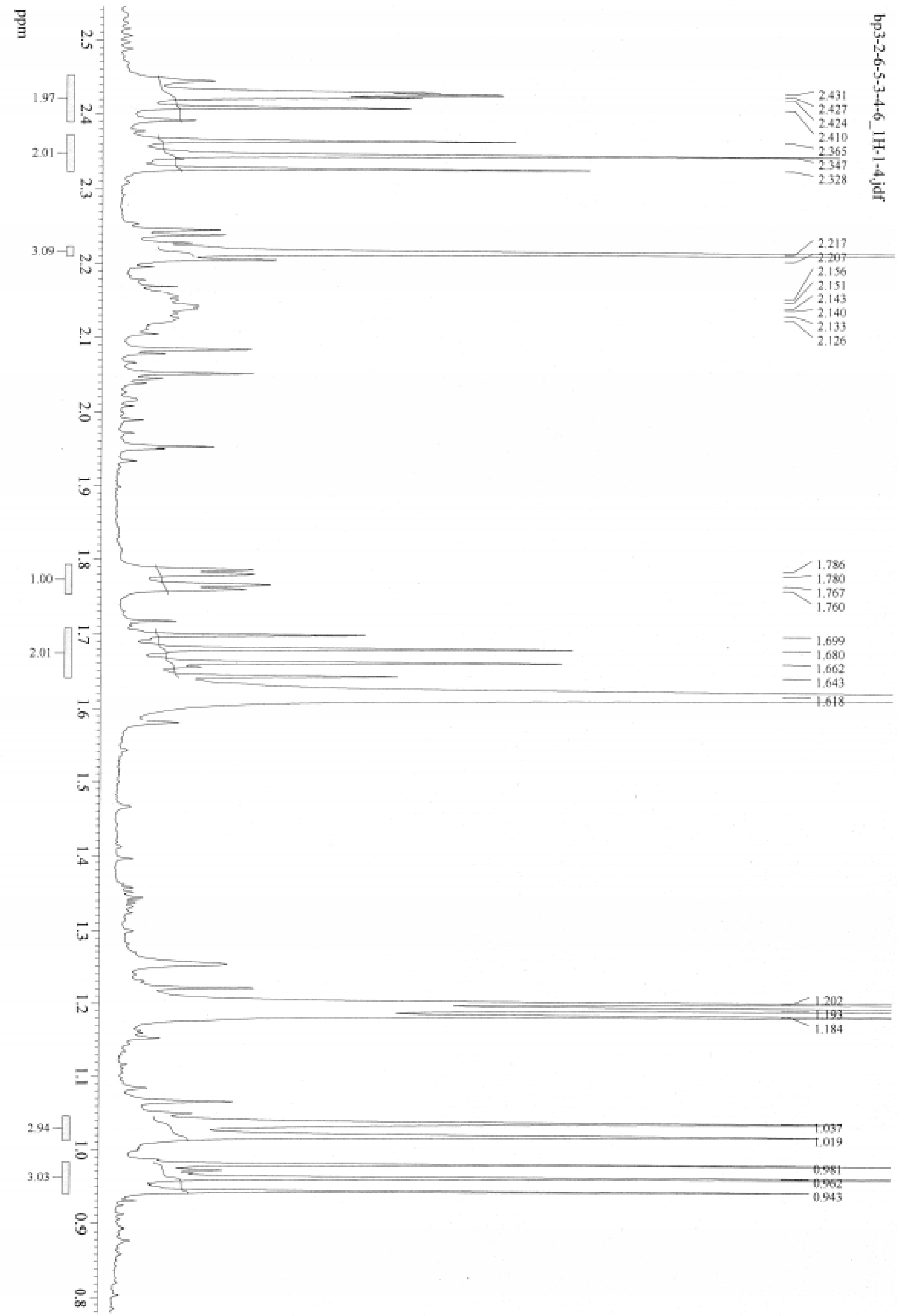

S27. Zoomed-in region of Figure S26 from 0.8-2.5 ppm

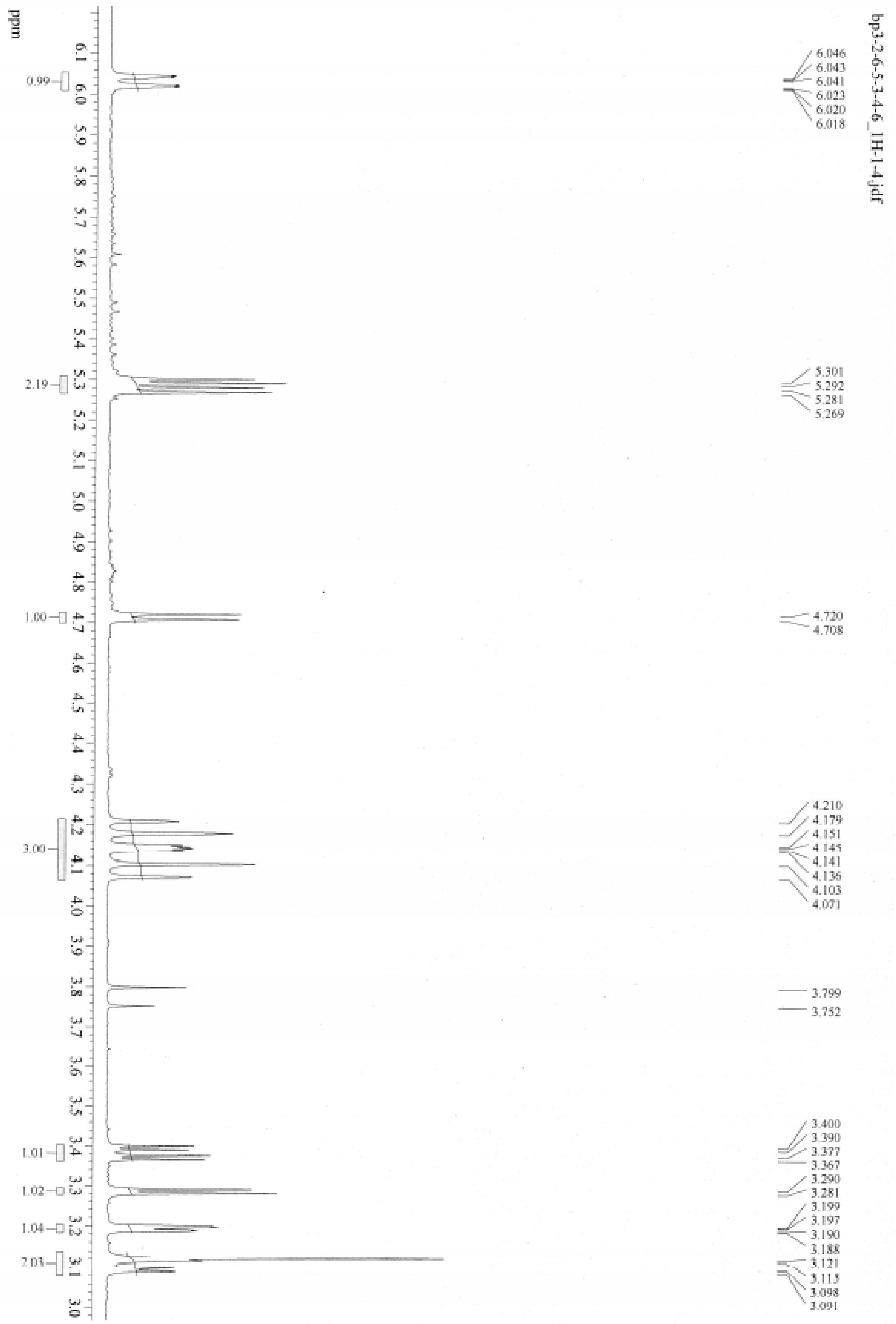

S28. Zoomed-in region of Figure S26 from 3.0-6.1 ppm

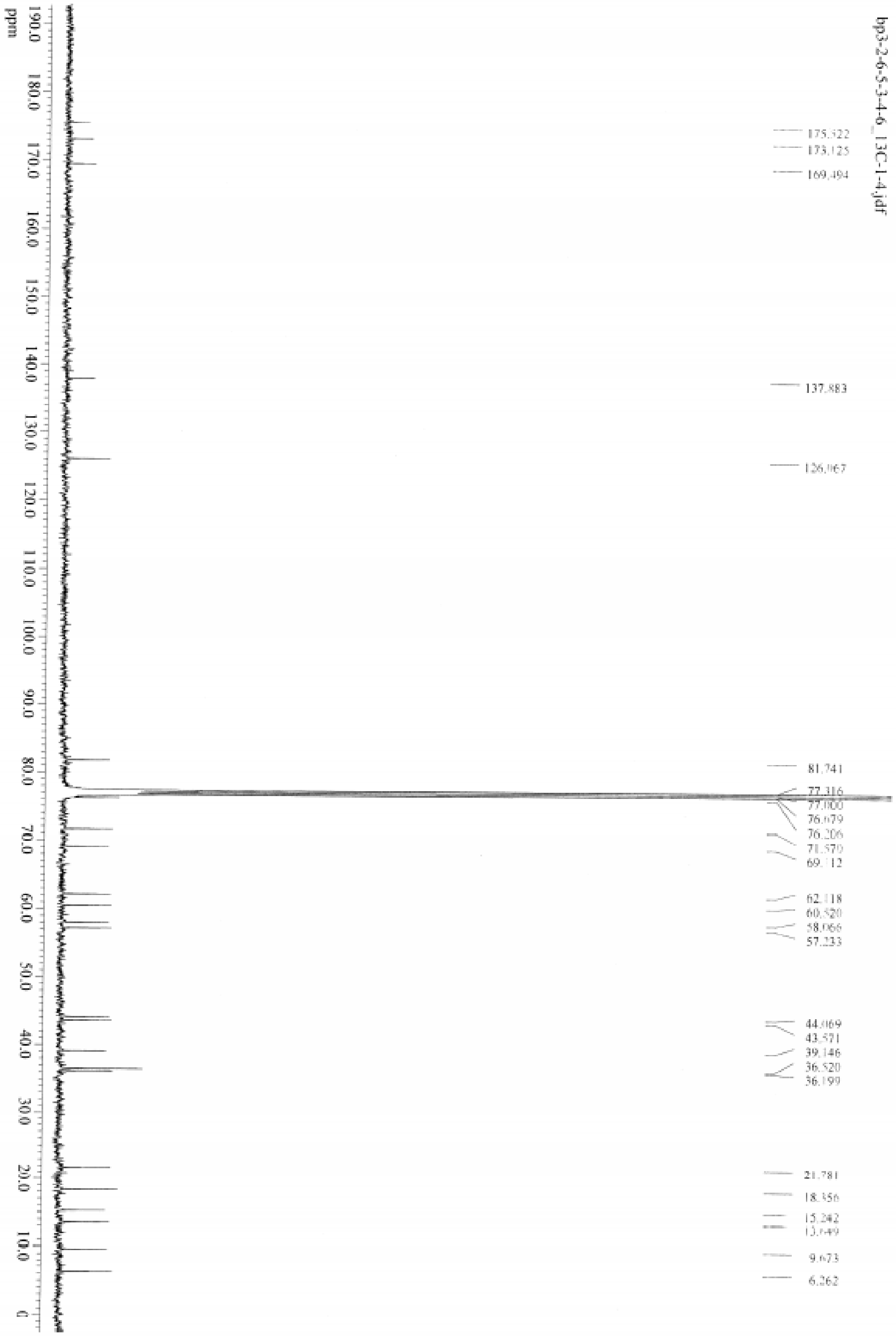

S29. ^{13}C NMR spectrum (150 MHz) of compound **3** in $CDCl_3$

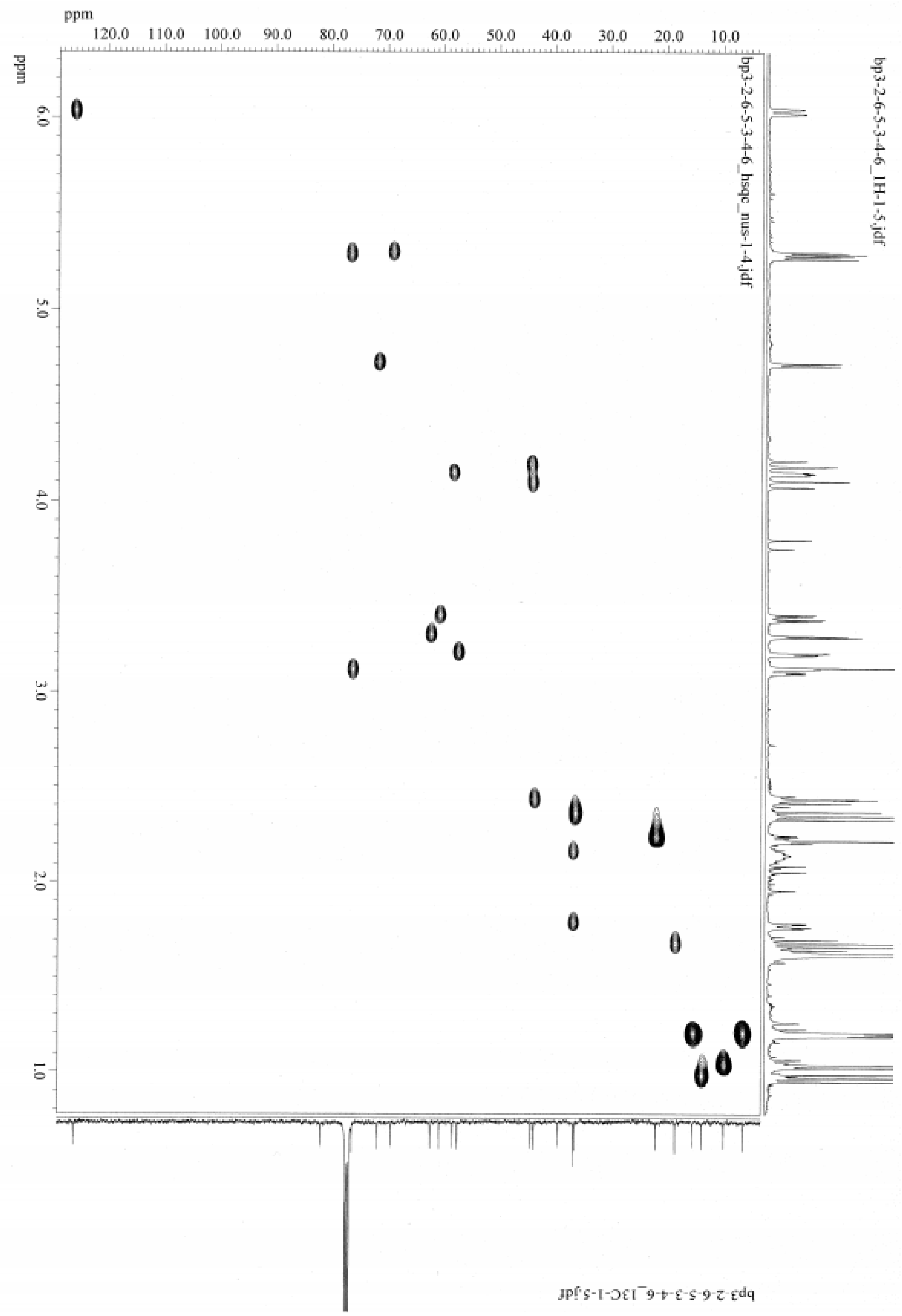

S30. HSQC spectrum of compound **3** in $CDCl_3$

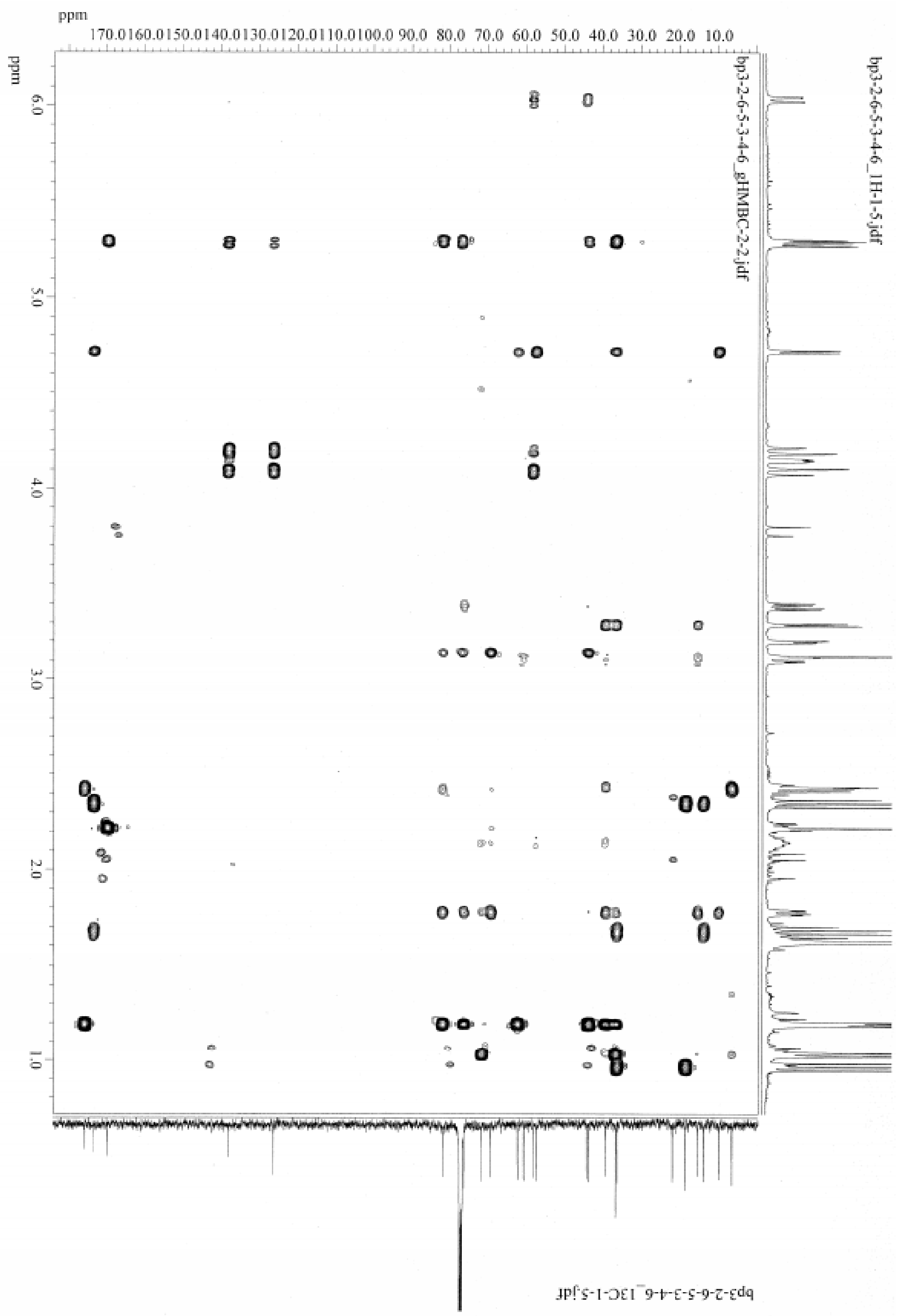

S31. HMBC spectrum of compound **3** in $CDCl_3$

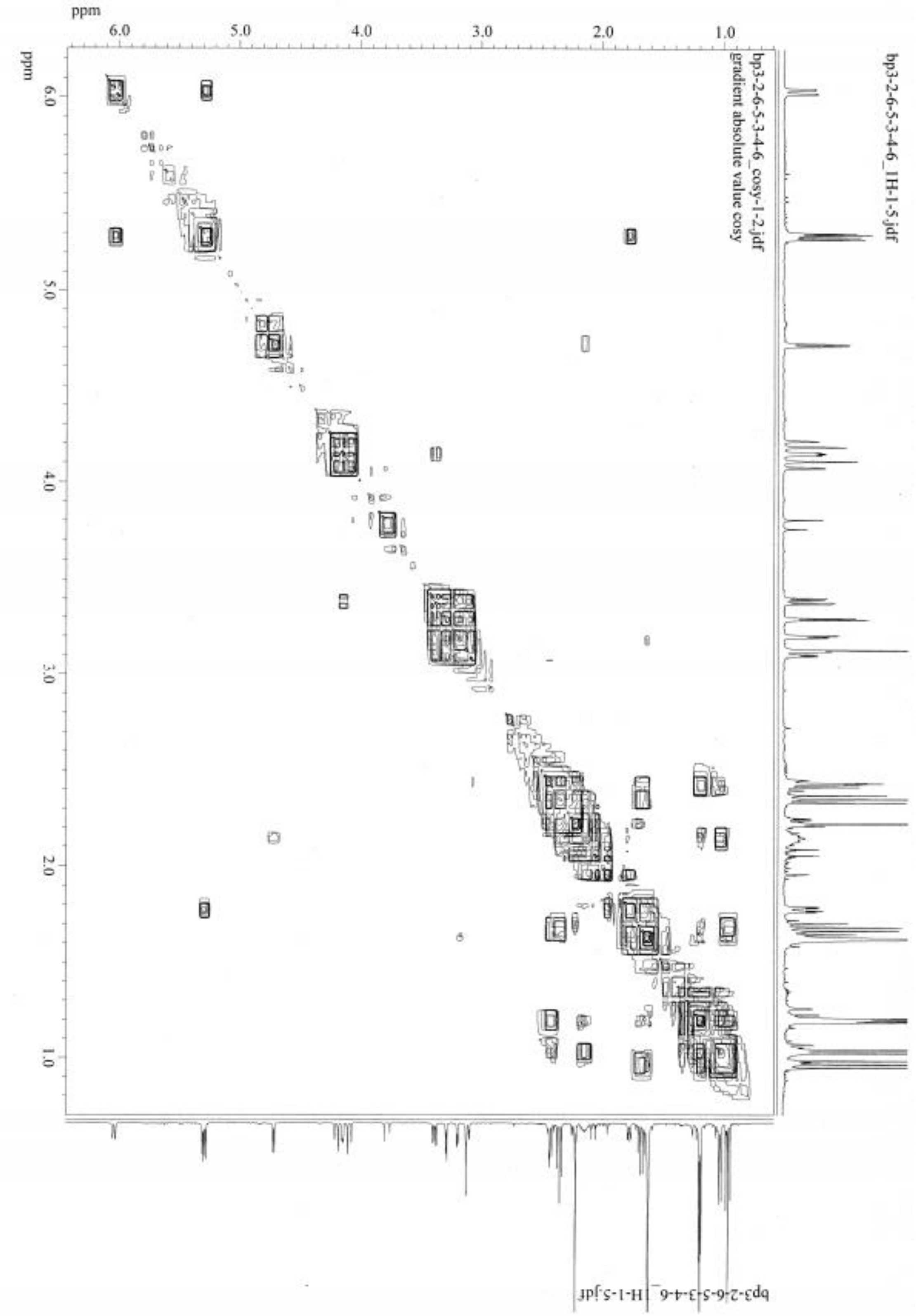

S32. ^{1}H-^{1}H COSY spectrum of compound **3** in $CDCl_3$

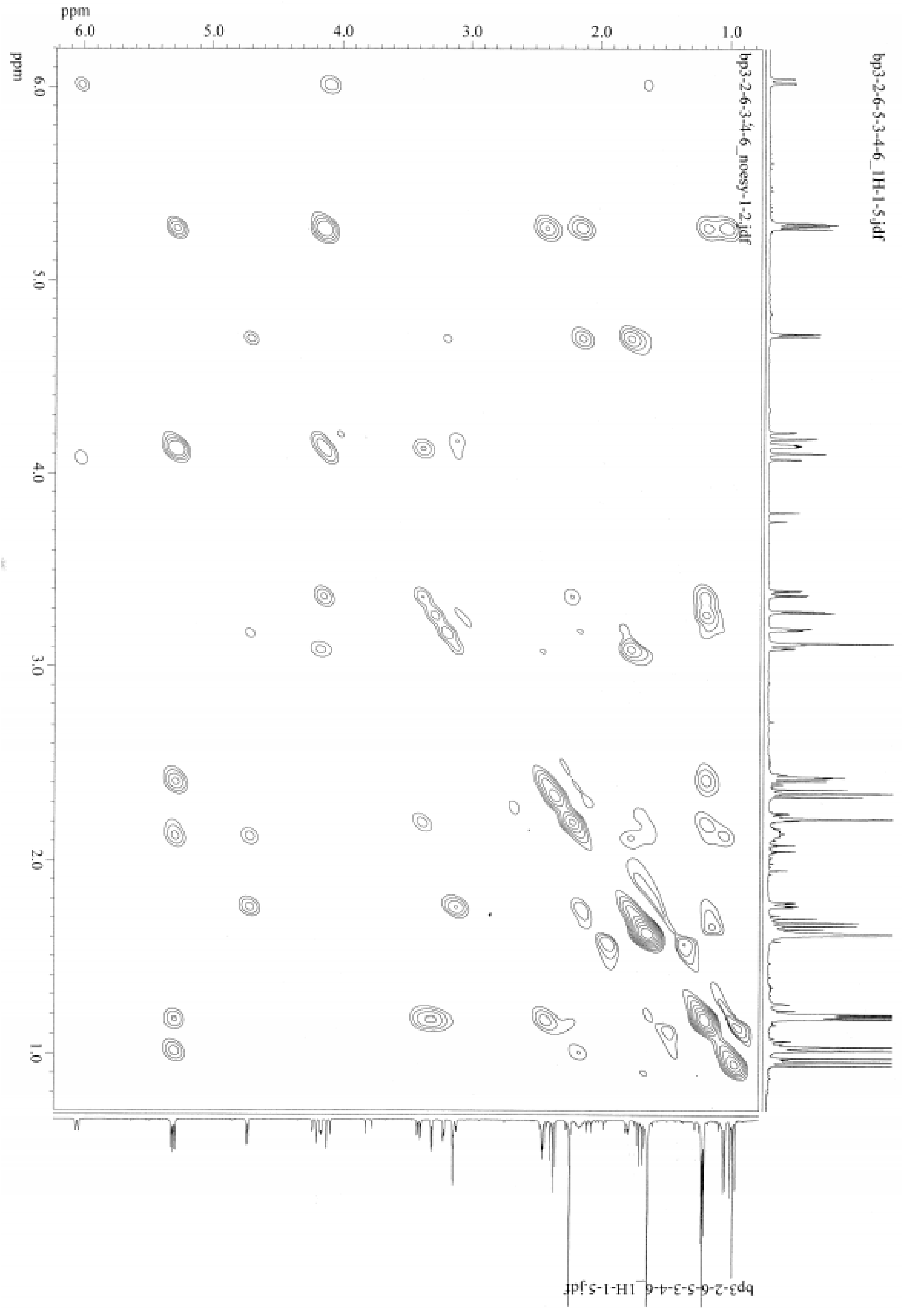

S33. NOESY spectrum of compound **3** in $CDCl_3$

FT-MS

Analysis Info

Analysis Name	D:\Data\a2\BP3265345_000041.d		10/15/2020 3:05:05 PM
Method	broadband first signal		
Sample Name	BP3-2-6-5-3-4-5		Instrument: FT-MS solariX
Comment	ESI Positive		

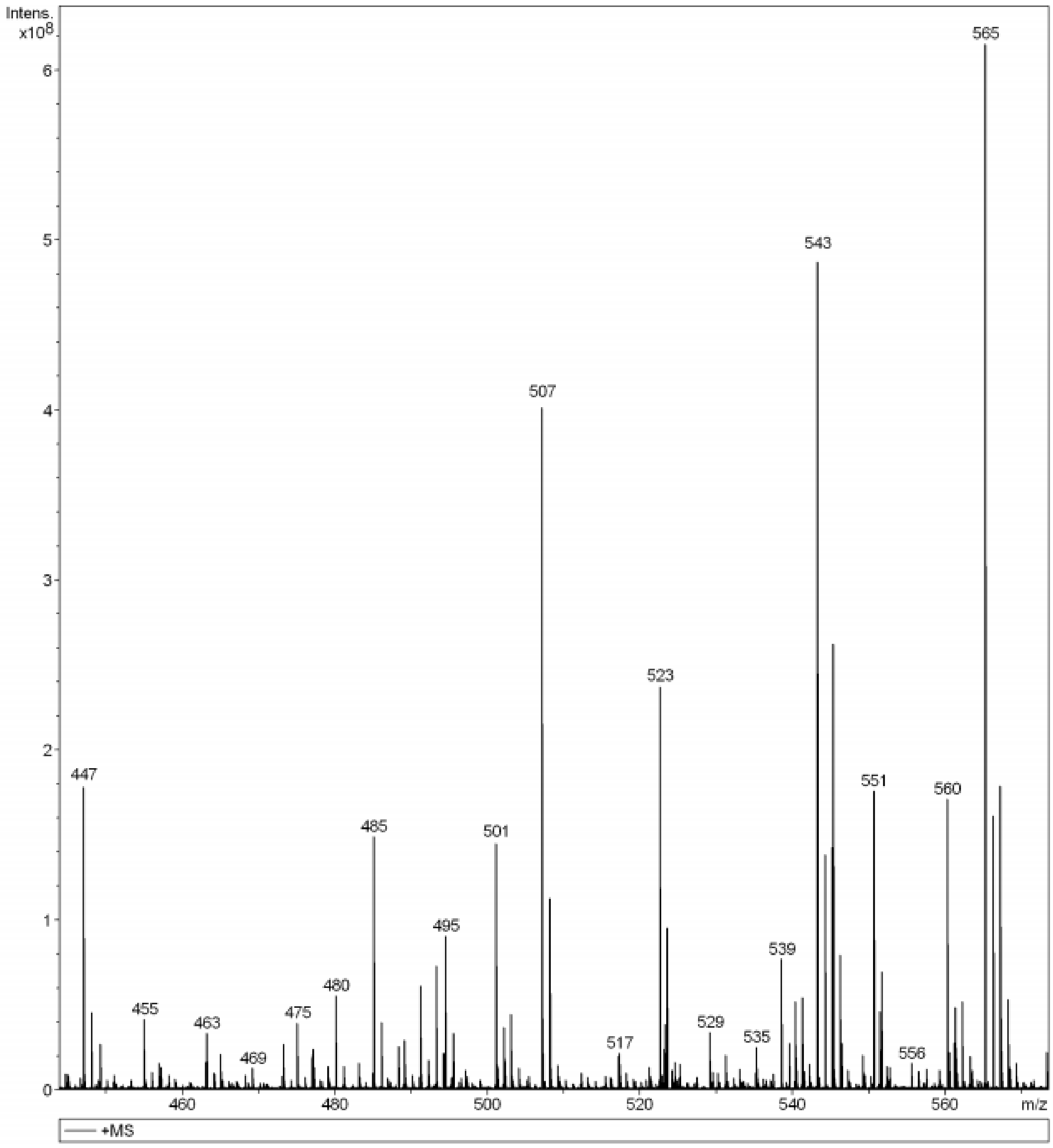

Bruker Compass DataAnalysis 4.0 printed: 10/15/2020 3:05:05 PM Page 1 of 1

S34. ESIMS spectrum of compound **4**

Mass Spectrum SmartFormula Report

Analysis Info

Analysis Name	D:\Data\a2\BP3265345_000043.d	10/15/2020 3:03:51 PM
Method	broadband first signal	Operator: YU HSIAO-CHING
Sample Name	BP3-2-6-5-3-4-5	Instrument: BRUKER FT-MS solariX
Comment	ESI Positive	

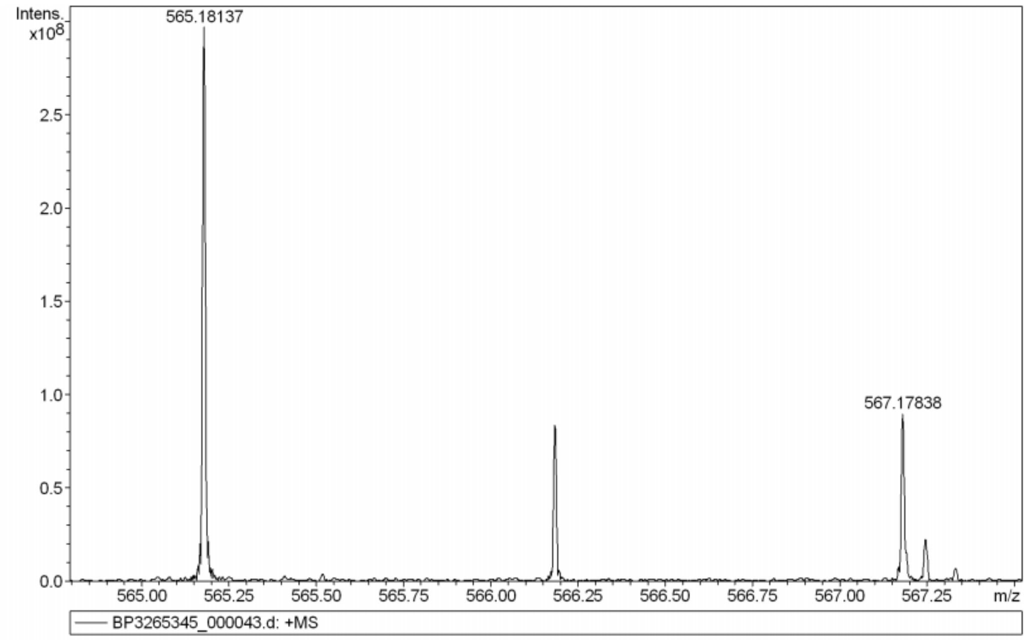

Meas. m/z	#	Formula	Score	m/z	err [mDa]	err [ppm]	mSigma	rdb	e⁻ Conf	N-Rule
565.18137	1	C 26 H 35 Cl Na O 10	100.00	565.18110	-0.27	-0.48	35.4	8.5	even	ok

S35. HRESIMS spectrum of compound **4**

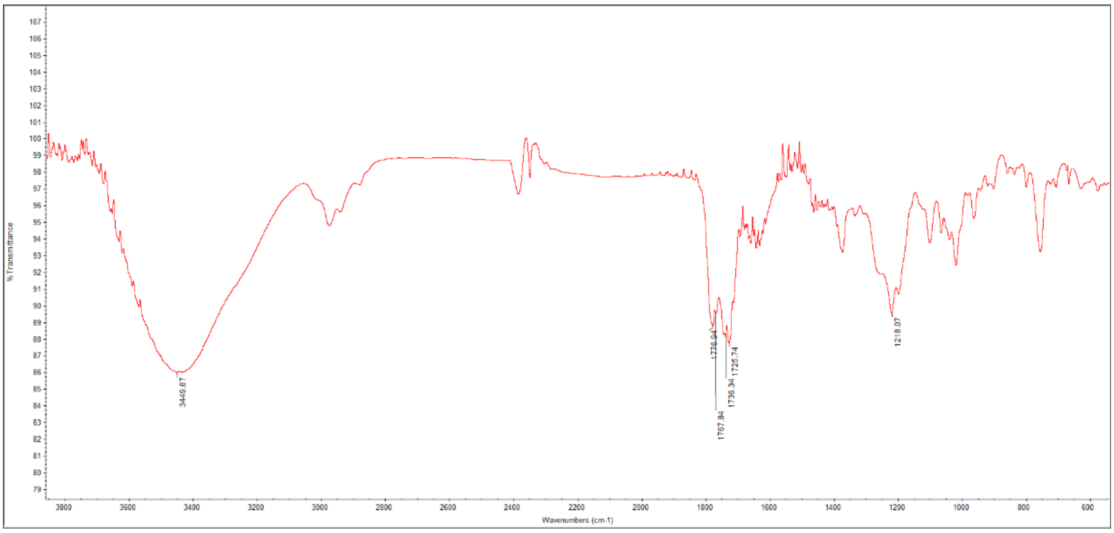

S36. IR spectrum of compound **4**

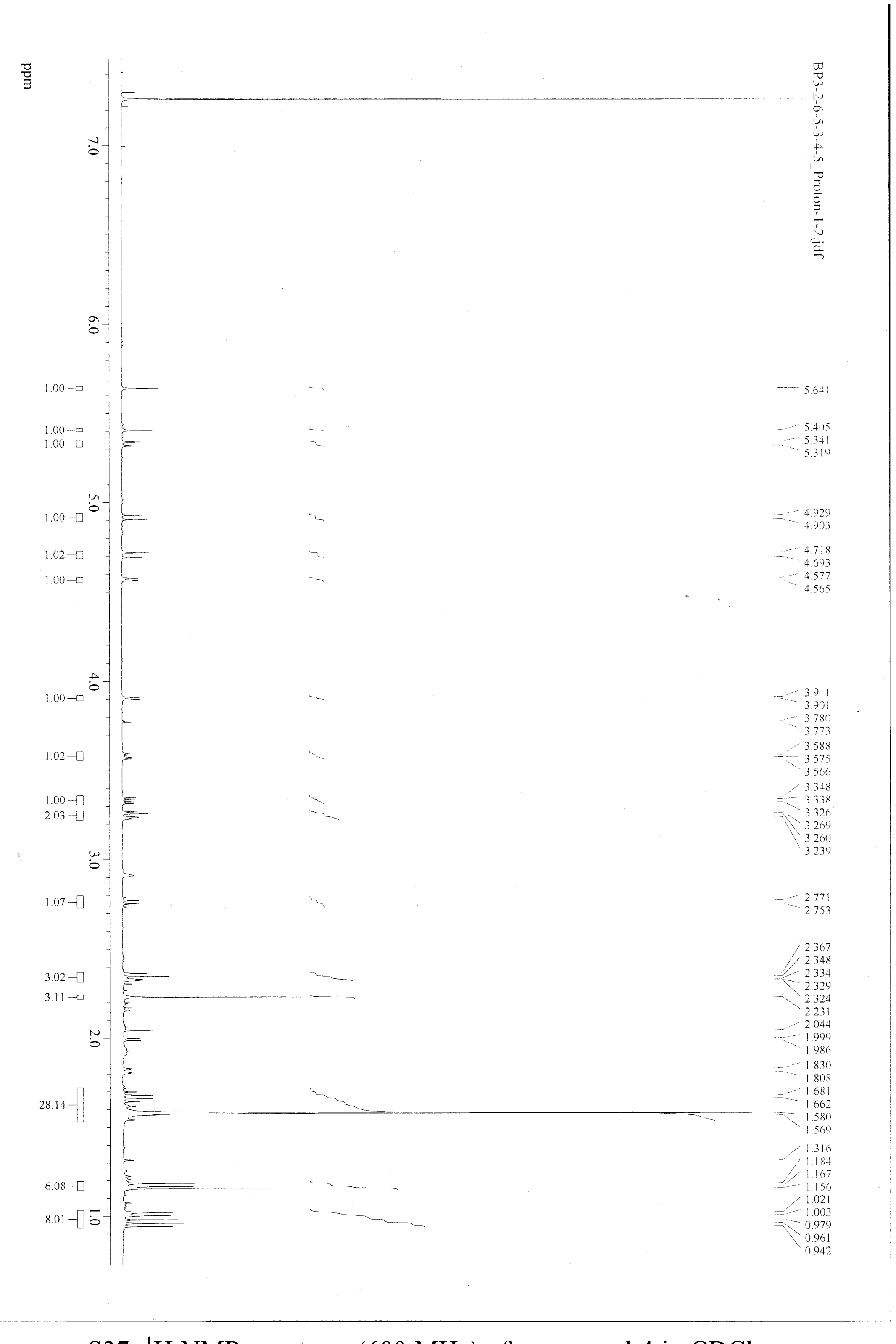

S37. ^{1}H NMR spectrum (600 MHz) of compound **4** in $CDCl_3$

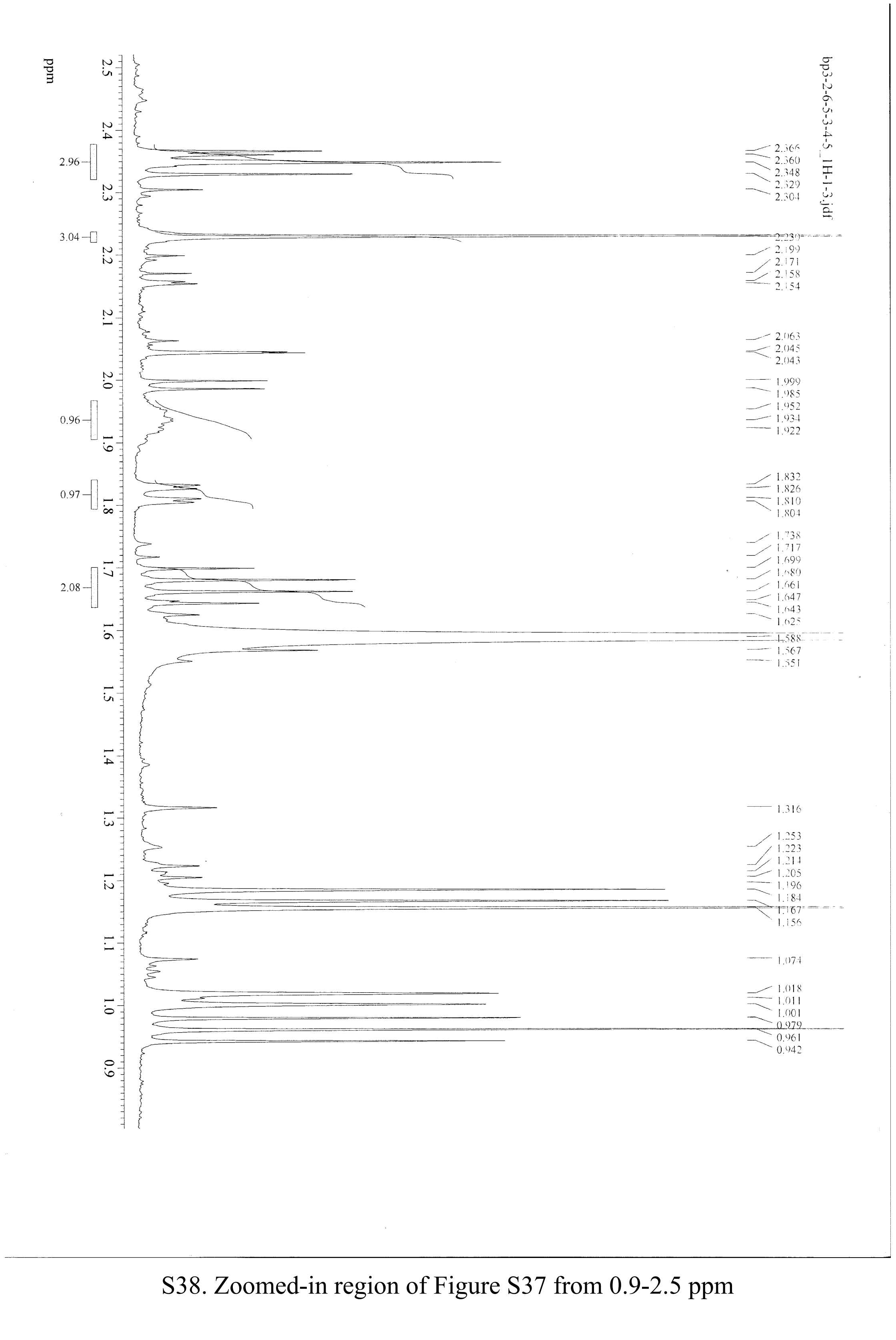

S38. Zoomed-in region of Figure S37 from 0.9-2.5 ppm

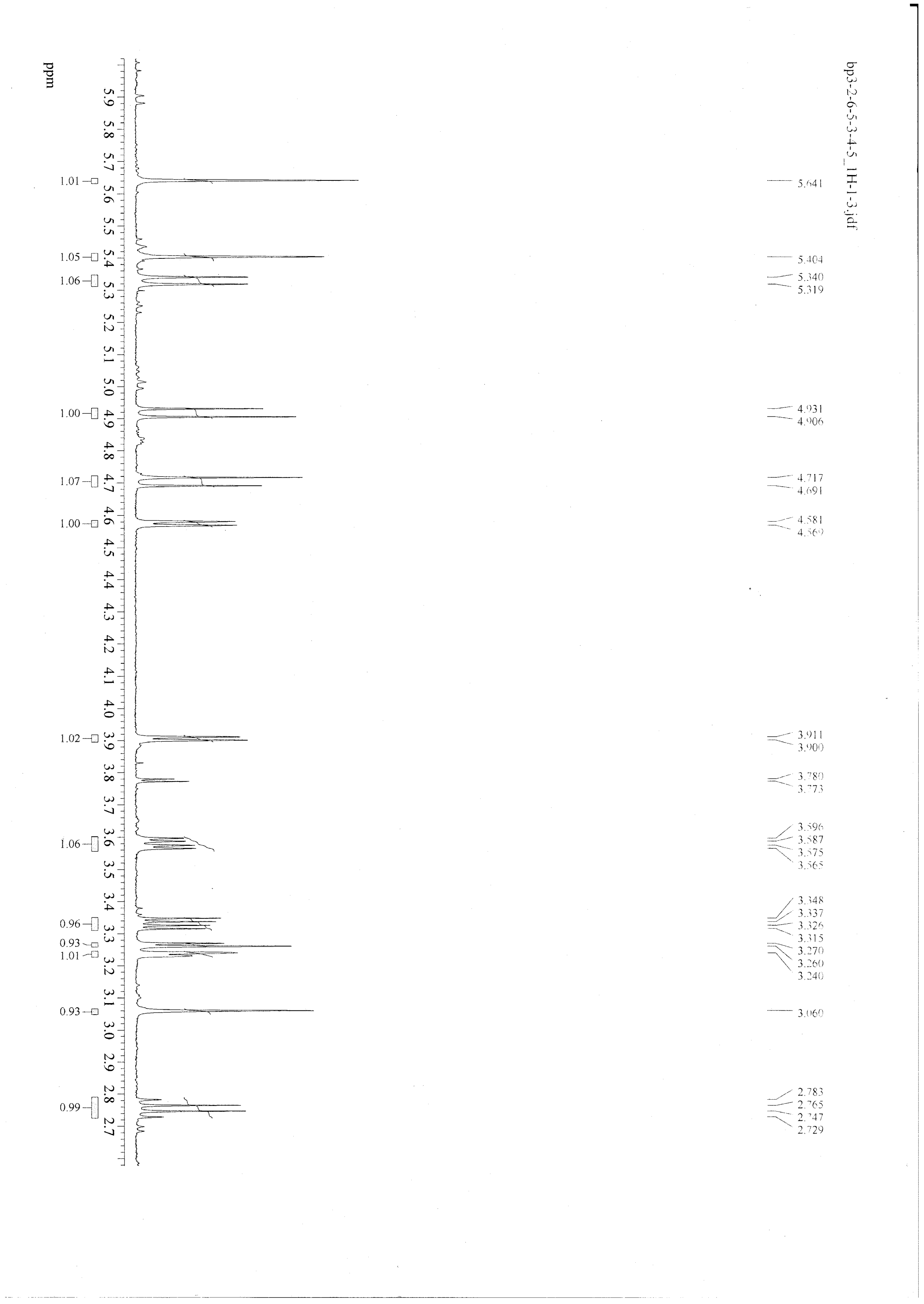

S39. Zoomed-in region of Figure S37 from 2.7-5.9 ppm

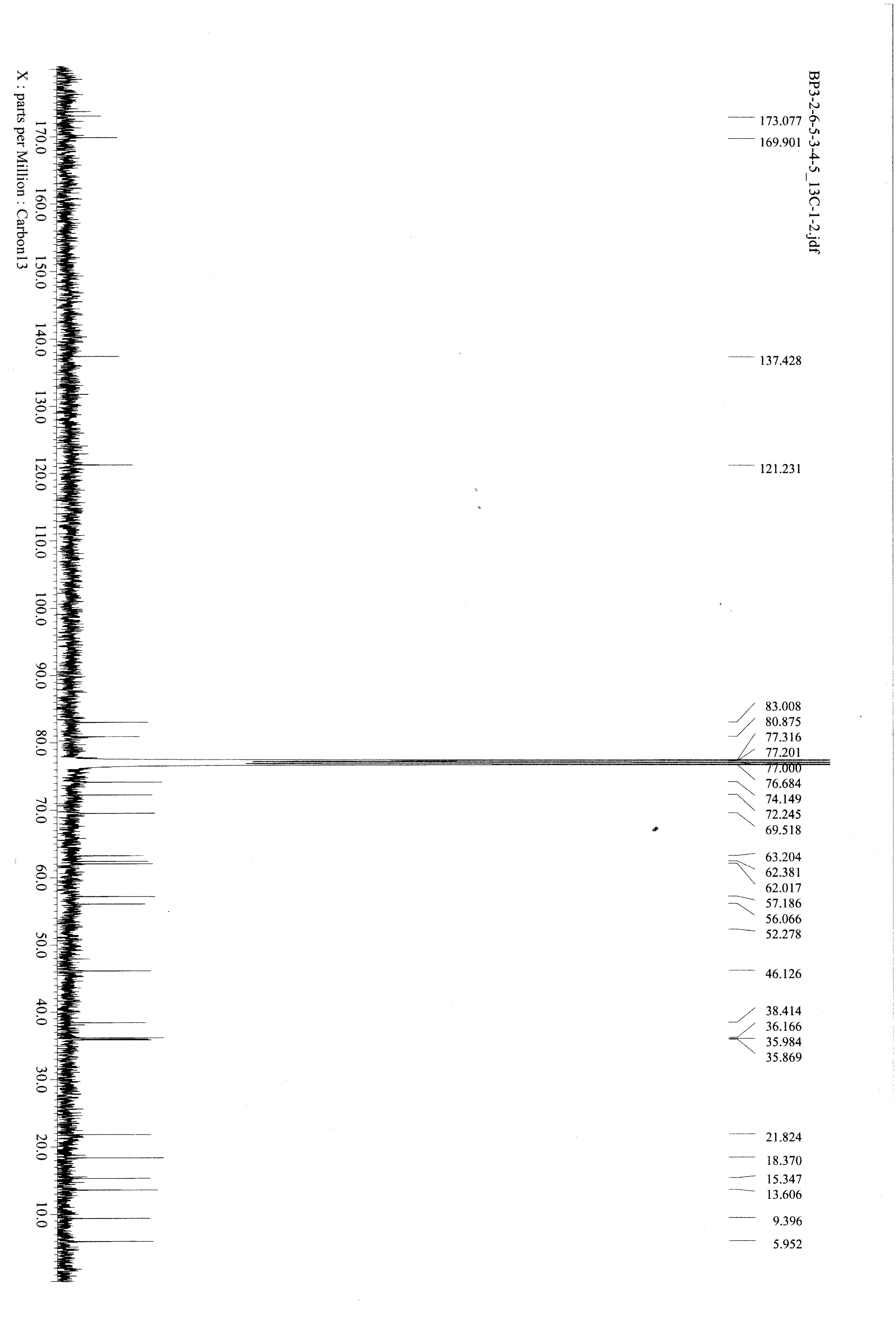

S40. ^{13}C NMR spectrum (150 MHz) of compound **4** in $CDCl_3$

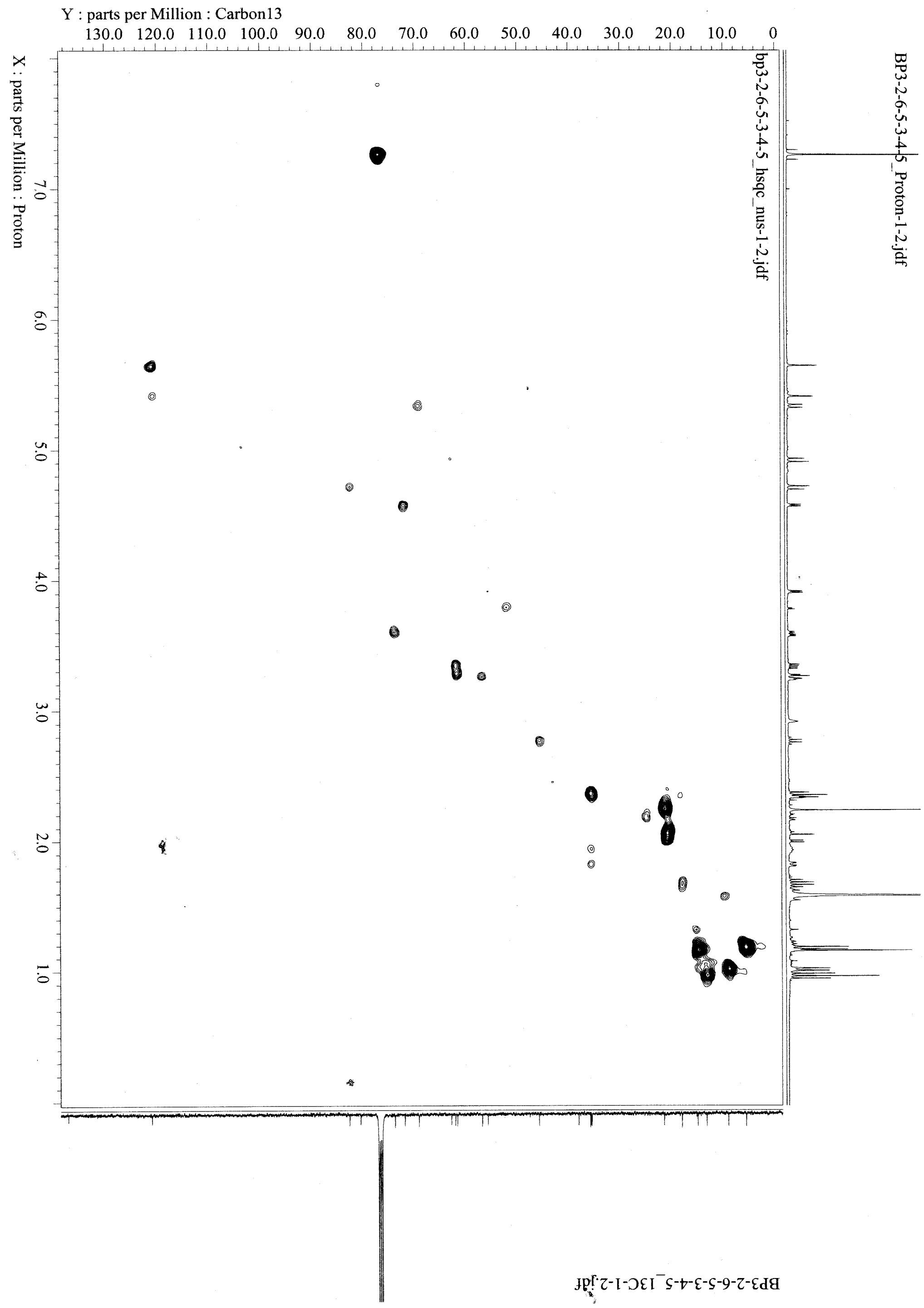

S41. HSQC spectrum of compound **4** in $CDCl_3$

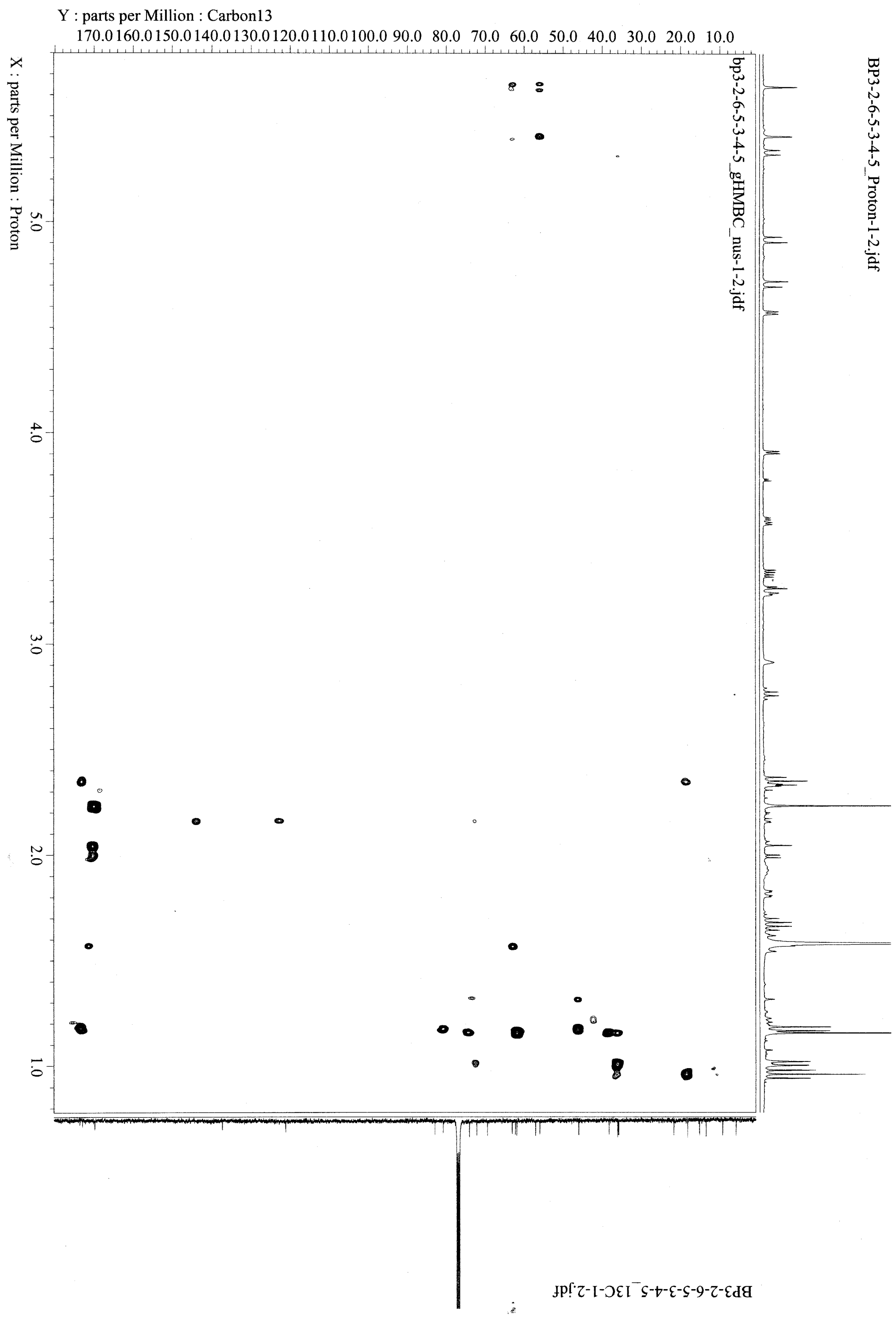

S42. HMBC spectrum of compound **4** in $CDCl_3$

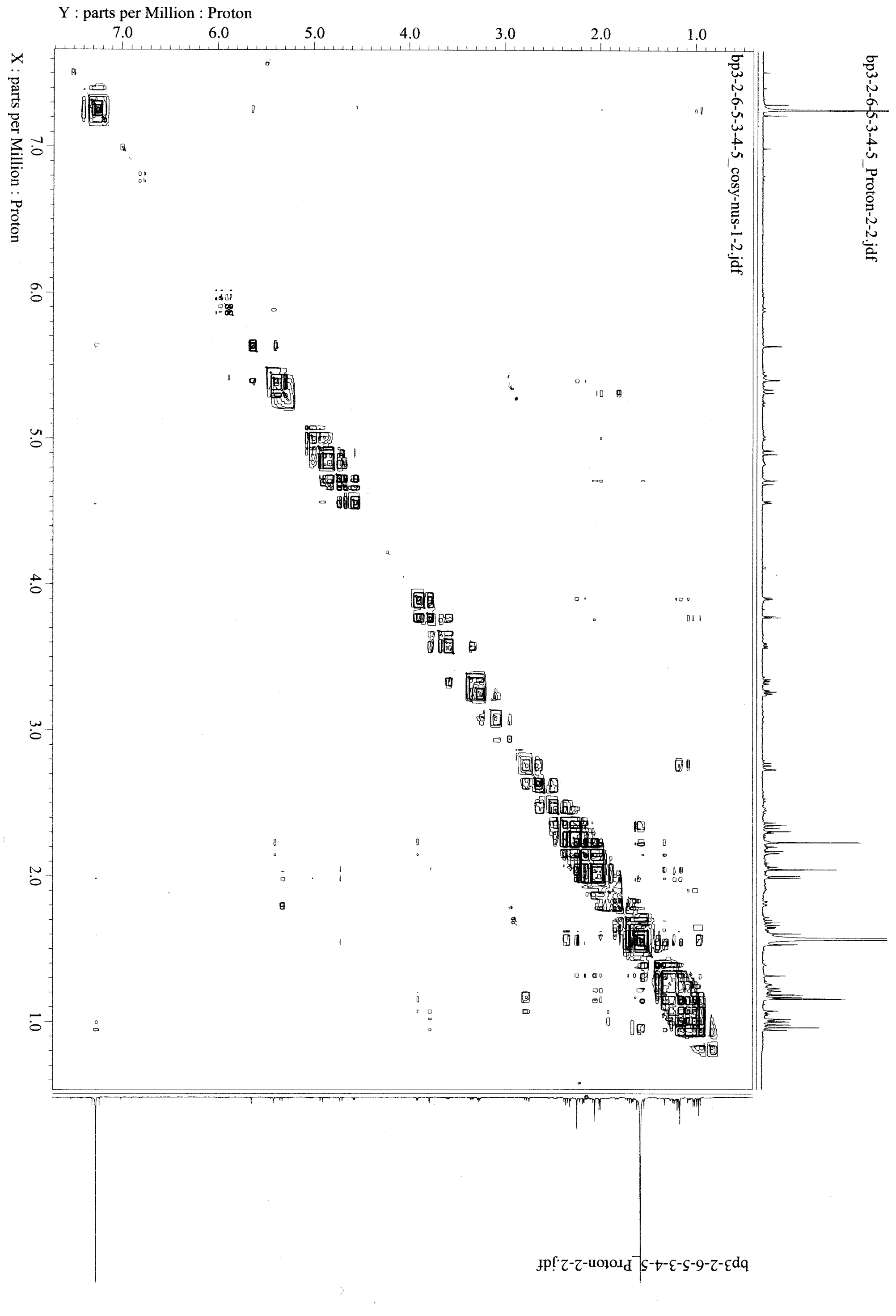

S43. ^{1}H-^{1}H COSY spectrum of compound **4** in $CDCl_3$

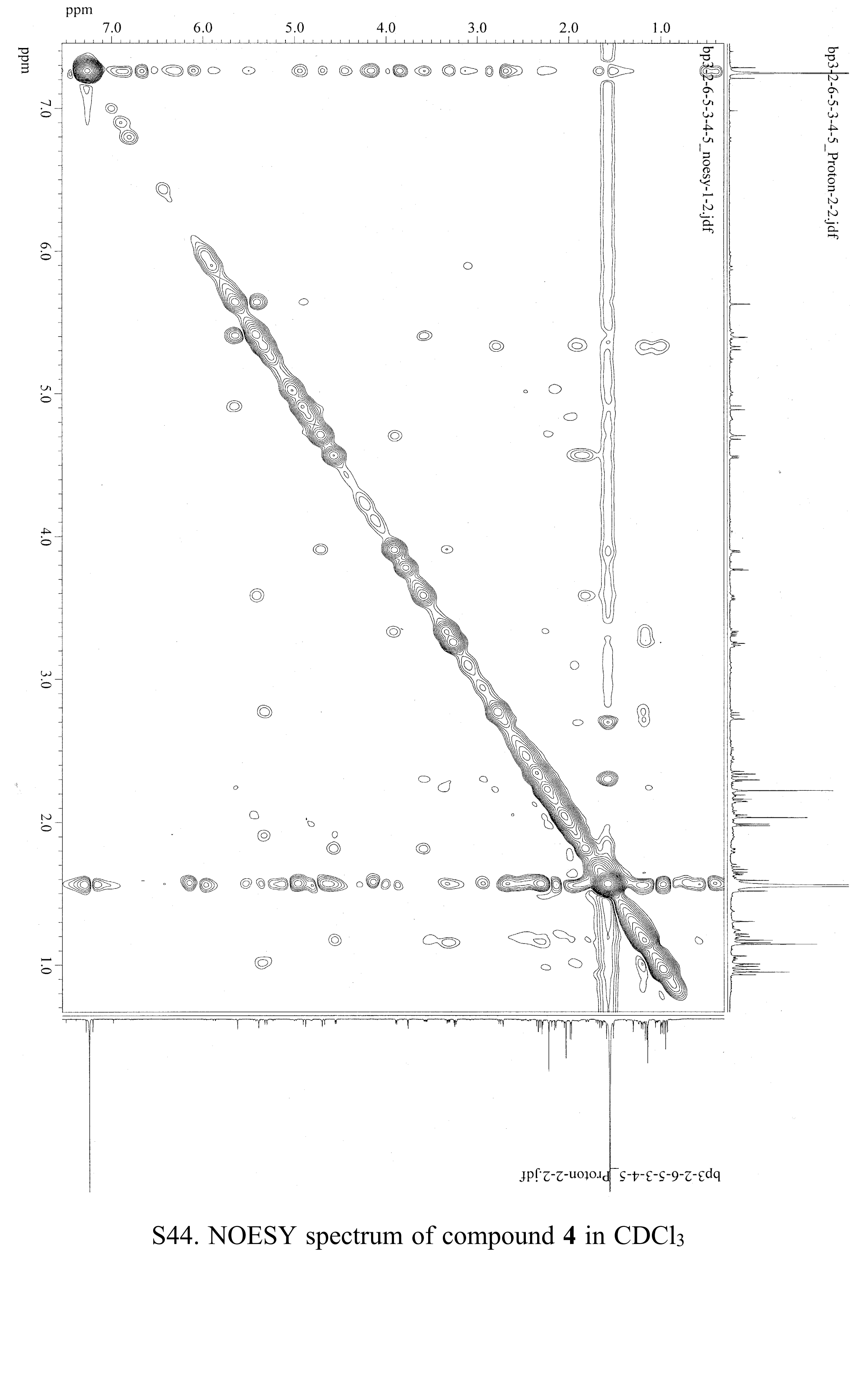

S44. NOESY spectrum of compound **4** in $CDCl_3$
